# Effect of the application of vermicompost and millicompost humic acids about the soybean microbiome under water restriction conditions

**DOI:** 10.3389/fmicb.2022.1000222

**Published:** 2022-11-04

**Authors:** Maura Santos Reis de Andrade da Silva, Lucas Amoroso Lopes de Carvalho, Lucas Boscov Braos, Luiz Fernando de Sousa Antunes, Camilla Santos Reis de Andrade da Silva, Cleudison Gabriel Nascimento da Silva, Daniel Guariz Pinheiro, Maria Elizabeth Fernandes Correia, Ednaldo da Silva Araújo, Luiz Alberto Colnago, Nicolas Desoignies, Everaldo Zonta, Everlon Cid Rigobelo

**Affiliations:** ^1^Programa de Pós-Graduação em Microbiologia Agropecuária, Universidade Estadual Paulista “Júlio de Mesquita Filho” (UNESP), Faculdade de Ciências Agrárias e Veterinárias, Jaboticabal, Brazil; ^2^Universidade Federal Rural do Rio de Janeiro (UFRRJ), Seropédica, Rio de Janeiro, Brazil; ^3^Faculdade de Ciências Agrárias e Veterinárias, Programa de Pós-graduação em Agronomia, Universidade Estadual Paulista (UNESP), Jaboticabal, Brazil; ^4^Departamento de Solos, Instituto de Agronomia, Universidade Federal Rural do Rio de Janeiro (UFRRJ), Seropédica, Rio de Janeiro, Brazil; ^5^Programa de Pós-graduação em Microbiologia Agrícola, Universidade Federal de Lavras (UFLA), Lavras, Minas Gerais, Brazil; ^6^Embrapa Agrobiologia, Seropédica, Rio de Janeiro, Brazil; ^7^Embrapa Instrumentação, São Carlos, São Paulo, Brazil; ^8^Phytopathology, Microbial and Molecular Farming Lab, Centre D’Etudes et Recherche Appliquée-Haute Ecole Provinciale du Hainaut Condorcet, Ath, Belgium

**Keywords:** humic substances, microbiome, inoculation, water stress, humic acid

## Abstract

Humic substances (HSs) are constituent fractions of organic matter and are highly complex and biologically active. These substances include humic acids (HA), fulvic acids (FA), and humin. HS are known to stimulate the root system and plant growth and to mitigate stress damage, including hydric stress. Humic acids have already been reported to increase microbial growth, affecting their beneficial effect on plants. However, there is scarce information on whether HA from vermicompost and millicompost, along with *Bradyrhizobium,* improves the tolerance of soybean to water restriction. This study aimed to evaluate the responses of soybean plants to the application of vermicompost HA (HA-V) and millicompost (HA-M) along with *Bradyrhizobium* sp. under water restriction. The experiment was carried out in a greenhouse, and the treatments received *Bradyrhizobium* sp. inoculation with or without the application of HA from vermicompost and millicompost with or without water restriction. The results showed that HA provided greater soybean growth and nodulation than the control. The application of HA-M stimulated an increase in the richness of bacterial species in roots compared to the other treatments. After the application of water stress, the difference between the treatments disappeared. Microbial taxa were differentially abundant in plants, with the fungal fraction most affected by HA application in stressed roots. HA-V appears to be more prominent in inducing taxa under stress conditions. Although the results showed slight differences between HA from vermicompost and millicompost regarding plant growth, both humic acids promoted an increase in plant development compared to the control.

## Introduction

Earthworm humus is recognized for its use in agriculture, as well as the humic acids (HA) extracted from this compound (García et al., 2018). The application of HA at low doses is capable of inducing beneficial effects on plants, improving their ability to take up nutrients and helping them overcome stress conditions (Canellas and Olivares, 2014). The role of HA in plant metabolism is closely related to the structure of these substances, and compounds from different matrices are expected to act differently in plants (Nardi et al., 2021). Another compound that has stood out for its benefit to plants is millicompost, which is produced from the processing of millipedes (*Diplopoda* sp.). These animals are able to use agricultural and urban waste, generating organic waste of great quality for plant growth. To date, there are no reports in the literature of the extraction and characterization of humic substances from millicompost.

The application of HA from different sources can increase soybean growth (Guo et al., 2015; da Silva et al., 2021a). This crop plays a key role in the world’s agricultural and economic scenario, and part of the success of soybean establishment in Brazil is due to the benefits of inoculating this crop with elite *Bradyrhizobium* strains (Hungria and Mendes, 2015). This symbiosis provides all the nitrogen needed for soybeans and reduces the dependence on nitrogen fertilizers and, consequently, prevents nitrogen losses by leaching and the emission of gases into the atmosphere (Hungria and Mendes, 2015). However, biotic and abiotic stresses can reduce crop productivity – even harming the contribution of biological processes to agriculture – and are therefore a risk to global food security (De Freitas et al., 2022). The application of agrochemicals to control environmental stresses may be ineffective, and in this context, the use of biostimulants has been recommended (Hossain et al., 2020). As HA plays an important role in plant resilience in relation to abiotic stresses and in the protection of bacteria in unfavorable environmental conditions, it is plausible that the use of HA contributes to the maintenance of biological processes even under adverse environmental conditions (Canellas et al., 2020; da Silva et al., 2021a).

It is known that the phytomicrobiome has the ability to act as an extension of the plant genotype (Carrión et al., 2019) and that its composition can be influenced by external factors such as the application of biostimulants (da Silva et al., 2021b). Changes in the plant microbiome can often increase tolerance to environmental stresses. Timm et al. (2018) demonstrated that different abiotic stresses have an impact on the bacterial community, enriching specific microbial groups, which may contribute to plant protection against adverse environmental conditions. A study showed that the application of a plant stress-related amino acid (1-aminocyclopropane-1-carboxylate, ACC) can remodel the soil microbiome and promote greater plant tolerance to salt stress (Liu et al., 2020). The application of HA to rice roots also caused changes in the composition of bacterial genera associated with the plant, and the authors suggested that the enriched bacterial groups would be related to plant protection; however, experiments under stress conditions have not been performed (da Silva et al., 2021b).

Despite this knowledge, there are practically no studies on the effects of HA on the community of endogenous and plant-associated bacteria and how this relationship affects plant growth, development and protection against biotic and abiotic stresses. It is well established in the literature that plants naturally harbor a great diversity of microorganisms in their tissues, and this microbiota is involved in functions that affect plant growth and survival (Vandenkoornhuyse et al., 2015). Therefore, the aim of this study was to evaluate the composition of the endophytic community of soybean plants in relation to the application of humic acids from different sources (vermicompost and millicompost) cultivated under different water availability conditions.

Specifically, the following hypotheses were tested: (i) the application of millicompost (HA-M) and vermicompost (HA-V) HA in soybean have different effects on plant growth and (ii) select different microbial groups related to plant growth promotion in water restriction.

## Materials and methods

### Extraction of humic acids

Millicompost and vermicompost came from an experimental agroecological farm, called “Fazendinha Agroecológica km 47”, which is a consortium between Embrapa Agrobiology, Federal Rural University of Rio de Janeiro and Pesagro. Humic acids and fulvic acids (FA) were extracted from these composts with KOH 0.1 mol L^−1^ in a ratio of 1:10 (v/v compost/solvent) under stirring for 4 h at room temperature. After that, the supernatant was separated from the humin (insoluble fraction), which was deposited at the bottom of the flask. Then, the supernatant (HA and FA) was acidified with 6 mol L^−1^ HCl to pH 1.5. Then, HA (insoluble fraction) was separated from FA by centrifugation at 2,000*g* for 10 min. The HAs were frozen and lyophilized.

### Characterization of humic acids

Humic acid samples were ground and sieved in sieves with a mesh size of 2 mm. Spectra acquisition was performed by cross polarization-magic angle spinning in a Bruker BioSpin 400 MHz spectrometer (Bruker Corporation, Billerica, United States) using a cross polarization-magic angle sample spinning sequence. Samples were placed in 4 mm probes using a rotor spinning speed of 14 kHz, and the spectra acquisition parameters were as follows: temperature of 297 K, ^13^C NMR frequency of 100.57 MHz, acquisition time of 0.04 s, contact time of 1 ms, recycle delay of 5 s, ^1^H and ^13^C pulse lengths of 2.5 and 3.8 μs, respectively, and 1,000 scans.

The spectra were processed with Bruker Topspin 3.2 software, phased, smoothed with a line broadening of 100 Hz, and baseline corrected. Each spectrum was then integrated after dividing it into chemical shift regions assigned to different carbon functional groups: 0–45 ppm (alkyl-C), 45–60 ppm (O- or N-substituted alkyl-C), 60–110 ppm (O-alkyl-and di-O-alkyl-C), 110–145 ppm (aromatic-C) 145–160, phenolic-C and 160–220 ppm (carbonyl-C + carboxyl-C). Based on the NMR results, the hydrophobicity index was calculated according to Aguiar et al. (2013).

The different carbon functional groups of humic acid samples were characterized using Fourier transform infrared (FTIR) spectroscopy on an IRAffinity-1 Shimadzu^®^ spectrometer according to the method described by Stevenson (1994). Measurements were performed using 1 mg of ground sample mixed with 100 mg of potassium bromide (KBr), operating between wavelengths from 400 to 4,000 cm^−1^. The elemental analyses of the C, H, and N contents of the HA were analyzed by a Perkin Elmer elemental analyzer (model 2,400). The results were as follows: C, 37.1%; H, 4.5; N, 3.1; C:N ratio, 12; H:C ratio, 0.120 for HA-M and C, 32.6%; H, 4.1; N, 3.3; C:N ratio, 10; H:C ratio, 0.125 for HA-V.

### Soil experiment

The experiment was set up in a randomized block design in a 3 × 2 factorial scheme, having as factors the application of humic acids and two water conditions (with and without water restriction). The experiment was carried out in a greenhouse using 5 L pots filled with red latosol, and fertilization was performed according to soil chemical analysis: pH, 6.9; C, 10 g kg^−1^; Al, 0 mmol kg^−1^; Ca, 79 mmol kg^−1^; Mg, 13 mmol kg^−1^; P, 14 mmol kg^−1^; K, 0.7 mmolc kg^−1^, no N source was added. Pots filled with dry soil were kept at field capacity, and irrigation, when necessary, was carried out with the same water volume for all pots until the time of water restriction. Treatments were composed as follows: (I) only inoculation of soybean seeds with commercial inoculant containing *Bradyrhizobium japonicum* SEMIA 5079 and *Bradyrhizobium diazoefficiens* SEMIA 5080; (II) inoculation of *B. japonicum* SEMIA 5079 and *B. diazoefficiens* SEMIA 5080 and application of 100 mg L^−1^ of millicompost HA in soybean seeds *via* the foliar route and (III) inoculation of *B. japonicum* SEMIA 5079 and *B. diazoefficiens* SEMIA 5080 and application of 150 mg L^−1^ of vermicompost HA in soybean seeds *via* the foliar route. A volume of 1 ml of inoculant was applied *via* soybean seeds of the ADV 4317 IPRO cultivar., and treatments that received HA had this compound added to the inoculant at the time of planting at the concentrations described above. The HA doses used in the present study were based on previous dose–response experiments of the different HAs in soybean. Each pot contained two seedlings, and foliar HA application occurred after 10 days of seed germination, with applications every 3 days. Three applications were performed at doses of 100 mg L^−1^ of millicompost HA and 150 mg L^−1^ of vermicompost HA, both diluted in water. In the treatment without HA, applications were performed only with water. After 25 days from the beginning of the experiment, a group of plants was submitted to water restriction for 5 days; in the other group with the same treatments, irrigation was maintained. Collection took place after 30 days of experimentation. The dry shoot and root mass, nodules and number of nodules, relative leaf water content (RWC) and chlorophyll and carotenoid contents were evaluated according to Wellburn (1994). Shoots and roots were collected for DNA extraction.

### Soil moisture, relative leaf water content and chlorophyll and carotenoid contents

Soil samples were collected at a depth of 10 cm, with three samples per pot, to obtain the soil moisture. The soil wet mass was weighed, and later, these samples were taken to an oven for 24 h at 105°C to obtain the dry mass. These measurements were used to obtain soil moisture based on the following equation: M% = [(soil wet mass) – (soil dry mass)]/(soil wet mass) × 100.

To evaluate the relative leaf water content, 15 disks were collected from the same leaf of the penultimate trefoil of each plant as a single sample; then, its fresh weight (FW) was measured. Subsequently, the material was submerged in distilled water for 6 h. The turgid weight (TW) of leaves was obtained, and after that, the material was placed in an oven at 60°C for 48 h to obtain the dry weight (DW). The following equation was used to calculate the RWC: RWC = [(FW – DW)/(TW – DW)] × 100%.

A total of 0.025 g of fresh leaf mass was removed from the penultimate trefoil and placed in tubes filled with 80% acetone. The extraction process was carried out in triplicate. After this period, readings were taken at the following wavelengths: chlorophyll a at 663 nm (A662); chlorophyll b at 647 nm (A645) and carotenoids (carotene [c] + xanthophylls [x]) at 470 nm (A470). To calculate the chlorophyll (Chl) and carotenoid (Car) concentrations, the formulas described by Lichtenthaler (1987) were used: Chl a + b = 7.05 A661.6 + 18.09 A644.8. Total chlorophyll (Chl a + b) and carotenoid contents were expressed as μg of pigment per gram of fresh mass (μg/g; Wellburn, 1994).

### DNA extraction

To obtain the endophytic community, shoots and roots were sterilized. First, the plant material was washed in running water and then added to sterile tubes containing 50% bleach (with 0.01% Tween 20). The plant material was vortexed at maximum speed for 2 min, the disinfectant solution was removed, and 70% alcohol was added. Subsequently, stirring was carried out at maximum speed for 1 min, and then, the alcoholic solution was removed and distilled water (previously sterilized) was added, again stirring for 1 min, which step was performed twice more. The water from the last wash was inoculated into Luria Bertani (LB) solid medium, and plates were incubated for 3 days at 28°C to infer the efficiency of the sterilization process. Sterilized shoots and roots were macerated with a previously sterilized mortar and pestle in the presence of liquid nitrogen, and 80 mg of the material was used for total DNA extraction. A Qiagen DNeasy^®^ PowerPlant^®^ kit was used, and the methodology was performed according to the manufacturer’s instructions. The quantity and quality of the total DNA extracted was evaluated in a NanoDrop^®^ ND 1000c spectrophotometer. The DNA concentration (ng μL^−1^) was measured by absorbance at a wavelength of 260 nm, and the relationship between absorbance values at 260 and 280 nm was used as an indicator of the extracted DNA quality. Integrity was assessed by 1% agarose gel electrophoresis. The gel was submitted to 70 V for 90 min and then stained with ethidium bromide and subsequently visualized with ultraviolet light in a transilluminator.

### Sample sequencing

For the sequencing analysis, three replicates of root and shoot DNA samples from each treatment were used, which were sent to be sequenced at the NGS company. The hypervariable region of the 16S rRNA V4 gene was amplified with the primers 515F (5′-GTGCCAGCMGCCGCGGTAA-3′) and 806R (5′-GGACTACHVGGGTWTCTAAT-3′; Caporaso et al., 2011), which were modified by adding degenerate nucleotides (Ns) in the 5′ region to increase the diversity of target sequences (de Souza et al., 2016). For the fungal fraction, the internal transcript spacer (ITS) was selected using ITS1f (CTTGGTCATTTAGAGGAAGTAA) and ITS2 (GCTGCGTTCTTCATCGATGC; Smith and Peay, 2014) primers. In both cases, library preparation followed previously established protocols with two-step amplification (de Souza et al., 2016; Armanhi et al., 2018). In the first PCR, the V4 prokaryotic region (16S rRNA) was amplified from the total DNA using the aforementioned primers with PNA-clamps (for the reduction of mitochondrial and chloroplast amplification; Lundberg et al., 2013). The products of this amplification were visualized on a 1.5% agarose gel. The purification of this PCR product was performed with Beckman’s AMPure XP beads. After purification, Illumina adapters were ligated into a PCR. These ligation products were further purified and then visualized on a 1.5% agarose gel. The adapter binding products were quantified and normalized to the same concentration. After normalization, an equimolar pool of each sample and qPCR quantification were performed to validate and determine the final pool concentration in nM. The kit used was the KAPA Library Quantification kit for Illumina. The equipment used was the Illumina MiSeq in 2 × 250 base pair (bp) runs.

### Analysis of experimentation data in the greenhouse

Experimentation data had normality and heteroscedasticity evaluated by the Shapiro–Wilk and Bartlett tests, respectively. Subsequently, analysis of variance (ANOVA) was performed, and when differences were detected, means were compared using Tukey’s multiple comparison test. There was no significant difference between the blocks; therefore, this effect was disregarded.

### Sequencing data processing

Processing started by assessing the quality of the data sequenced using the “FastQC” software (v.0.11.9). Subsequently, the cut-off parameters of the sequences were established. For this, the “-fastx_info,” “-fastq_eestats2” and “-search_oligodb” functions from the “USEEARCH” software (v.11.0.667) were used, where the average quality thresholds and size and placement of adapters were obtained. Adapters were then removed using the “atropos” software (v.1.1.21), where sequences that did not contain them (“--discard-untrimmed”) or were smaller than 200 bp (“--minimum-length = 200”) were discarded. Adapter-free sequences underwent a quality control process using the “PRINSEQ-lite” software (v.0.20.3), where the final end was excluded, whose quality window (“-trim_qual_window = 3”) was <20 and 18 for the “forward” (R1) and “reverse” (R2) sequences of each library, respectively. Finally, library pairs were sorted (“fastq_pair”; v.1.0) and merged (“PEAR”; v. 0.9.11). The merged sequences were submitted to the “DADA2” pipeline (v.1.22.0) through a package made available for R statistical software (v. 4.1.2; R Core Team, 2021), for the establishment of amplicon sequence variants (ASVs). The pipeline starts with a quality control (“filterAndTrim”), where sequences were filtered and truncated respecting the insert size of the 16S rRNA gene V4 region (“truncLen = 250”) – for ITS sequences, whose size shows great variability, this parameter was suppressed. Then, possible sequencing errors were identified (“learnErrors”) and corrected to obtain the exact ASV for each sample. Each ASV was taxonomically classified (“assignTaxonomy”) based on the SILVA (v.138.1) and UNITE (v.8.2) reference banks for 16S rRNA and ITS libraries, respectively.

### Diversity analysis and statistics

ASV counts per sample, as well as the relative taxonomic classifications, were imported as a “phyloseq” object (R package “phyloseq”; v.1.38.0) transformed into compositional data by the function “phyloseq_standardize_otu_abundance” (R package: “metagMisc” – v.0.0.4) with the “total” method, which applies total sum scaling (TSS) to the dataset. For alpha diversity, richness (Chao1) and diversity measures (Shannon and Gini-Simpson indices) were calculated using the “alpha” function of the “microbiome” R package (v.1.10.0). The existence of general differences in the alpha diversity means was evaluated with ANOVA/Kruskal–Wallis tests (depending on data distribution), and in the case of significance (*p*-value < 0.1), means were compared pair-to-pair with Student/Wilcoxon *t*-tests. Additionally, we performed a three-way ANOVA with alpha diversity measures to assess whether the interaction between the evaluated factors could influence these measures. For beta diversity, dissimilarities were calculated using the Bray–Curtis index (“distance” function of the “phyloseq” R package), from which the hierarchical clustering of samples was extracted in the form of a dendrogram, as well as a principal coordinate analysis (PCoA). The statistical significance of the separation of conditions evaluated was given through PERMANOVA, considering a *p*-value of 0.1. Finally, microorganisms that had significant changes in abundance among the conditions studied were identified. For this, the “DESeq2” approach was used, which implements the Wald test for the comparison of means (*p*-value < 0.05). For this analysis, samples were sectioned concerning plant tissue + stress condition and compared to humic acid treatments (formula = “plant tissue + stress ~ treatment”). The microbiota was collapsed at each taxonomic level and compared with the raw count sum of each taxon.

## Results and discussion

Regarding the variables soil moisture, RWC and pigment content, only the stress factor differed (*p*-value ≤ 0.05). Soil moisture, RWC and pigment content were significantly higher in irrigated plants than in plants submitted to water restriction. Regarding plant dry mass, differences were observed between treatments with HA in both water conditions (Table 1).

**Table 1:** Soybean plant parameters evaluated under different water conditions.

	Shoot mass (g)		
	Without stress	With stress	
HA			Overall average
HA-V	7.86Aa	3.37Aa	5.94A
HA-M	8.01Aa	3.65Aa	6.07A
Control	4.65Ba	0.82Ab	3.18B
Overall average	6.40a	2.40b	
	**Root mass (g)**		
HA			Overall average
HA-V	1.46Aa	1.23Aa	1. 36A
HA-M	1.31Aa	0.88Aa	1.12AB
Control	0.89Aa	0.39Aa	0.70B
Overall average	1.15a	0.76a	
	**Nodules mass (g)**		
HA			Overall average
HA-V	0.43Aa	0.11Ab	0.29A
HA-M	0.30Ba	0.15Ab	0.23A
Control	0.07Ca	0.02Aa	0.06B
Overall average	0.22a	0.09b	
	**No. of nodules**		
HA			
HA-V	127.0Aa	44.25Ab	87.85A
HA-M	80.40Ba	35.65ABb	64.33B
Control	29.37Ca	6.00Ab	20.38C
Overall average	67.35a	26.16b	

Means followed by different letters, uppercase in the column compares HA-V with HA-M and with the control (Humic acid-HA), lowercase in the row compares the treatment with and without stress and differ from each other by the Tukey’s test at 10% significance level.

In the present work, millicompost and vermicompost HA were evaluated, and to date, we have no reports of studies that have extracted and characterized millicompost HA and evaluated its effect on plants and their microbiota. The effect of millicompost and vermicompost HA on soybean growth was compared with that of the control (treatment that did not receive HA), both under favorable environmental conditions and under water deficit (Table 1).

Nuclear magnetic resonance and FTIR analyses revealed structural differences between HA-M and HA-V (Table 2; Supplementary Figure S1). These differences result from the biological and biochemical composting processes, in addition to differences in the composition of the source material itself. The ^13^C NMR results show that both composts were in an advanced stage of humification with strong C aromatic and phenolic groups (Tadini et al., 2022). The HA-M and HA-V aromatic C represent ~24 and 20.3% and C-Phenolic ~10 and 7.1% of the total area NMR spectra, respectively. The higher aromatic and phenolic C contents in HA-M than HA-V may indicate differences in the original organic material sources or differences in the composting process. These differences in chemical composition might help to explain the differences related to plant performance (Table 1). HA-M has a lower proportion of C-alkyl groups, which are organic groups formed by saturated hydrocarbons and are relatively inert in the soil and contribute to increasing the hydrophobicity index (Table 2). Both composts had similar levels of C-alkyl-O (Table 2), revealing that most of their bioavailable molecules were consumed during the stabilization process.

**Table 2:** Distribution of different types of carbons, relative areas of spectral regions and the degree of aromaticity in humic acids.

	C-alkyl	C-alkyl-N/methoxyl	C-alkyl-O	C-aromatic	C-carbonyl/amide	Hydrophobicity Indices	C-Phenolic
	0–45	45–60	60–110	110–140	160–220		145–160
HA-M	23.0%	11.4%	22.7%	24.0%	8.9%	1.38	10.0
HA-V	30.5%	13.1%	23.1%	20.3%	5.9%	1.74	7.1

HA-M, Millicompost humic acids; HA-V, Vermicompost humic acids.

The FTIR spectra of HA-M and HA-V are similar, indicating similar chemical compositions, with the exception of the peak from 1,200 to 950 cm^−1^, which is stronger in HA-V than in HA-M (Supplementary Figure S1). The absorbance from 3,000–3,500 is assigned to OH groups of phenolic and carboxylic acids and alcohols and NH groups of amines and amides, from 3,000 to 2,800 is assigned to C-H alkyl bonds and from 1,800 to 1,550 is assigned to C=O bonds of carbonyl, quinone, carboxylic acids, esters and amide, and the C=C vibration of aromatic molecules shows similar intensities. The band between 1,200 and 900 cm^−1^ is stronger in HA-V, indicating a larger content of C-O bonds (Osiro et al., 2004) than in HA-M, which might be due to the residual polysaccharides and lignin phenolic and O-CH_3_ groups.

The concentration of millicompost HA (100 mg L^−1^) was lower than that of vermicompost HA (150 mg L^−1^), providing similar results on plants. These results may indicate that millicompost HA is more bioactive in plants than vermicompost HA, and this effect is possibly related to the chemical composition of these substances, as seen in Table 2, which, in turn, was influenced by the compound matrix, given that the extraction method was the same for both.

Supplementary Table S1 shows the counts of 16S rRNA amplicon reads throughout data processing per sample (see Supplementary material). Supplementary Table S2 shows the counts of ITS amplicon reads throughout data processing per sample. Supplementary Table S3 shows the counts of 16S rRNA amplicon reads throughout data processing, per group. Supplementary Table S4 shows the counts of ITS amplicon reads throughout data processing, per group. Supplementary Table S5 shows the counts of 16S rRNA amplicon reads classified in each taxonomic rank, and Supplementary Table S6 shows the counts of ITS amplicon reads classified in each taxonomic rank.

**Figure 1:**
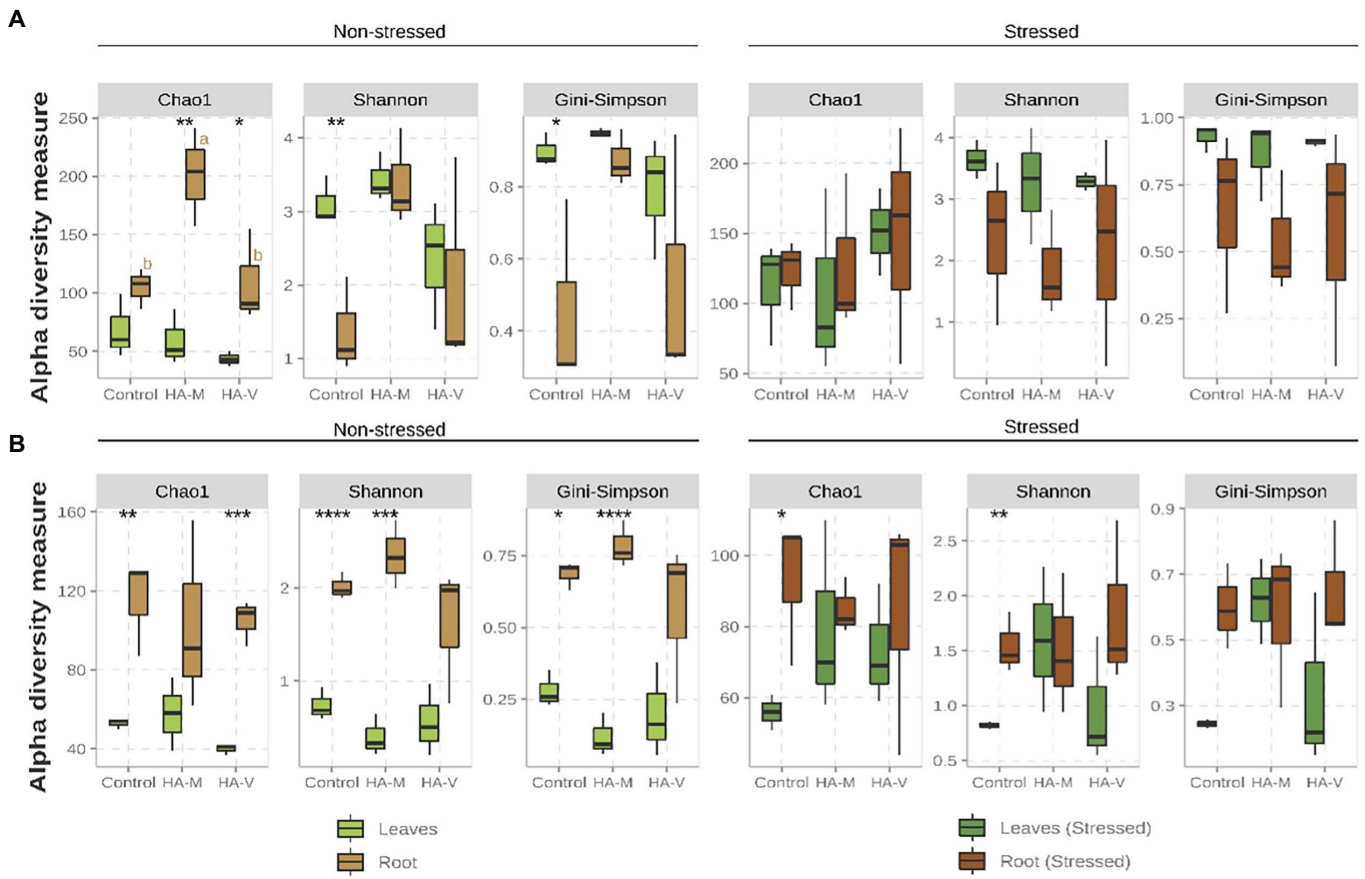
Boxplot of the alpha diversity metrics of bacterial **(A)** and fungal **(B)** fractions present in the shoot and root of soybean plants that received the application of vermicompost HA (HA-V), millicompost HA (HA-M) or that did not receive application (Control) in the presence and absence of stress. Different letters indicate significant differences between HA application within the same plant material (*post-hoc* test). Significant differences between different plant tissues under the same conditions (HA application and presence or absence of stress) can be seen by the presence of the following symbols: ****p < 0.001; ***p ≤ 0.01; **p ≤ 0.05; *p ≤ 0.1. The absence of letters/symbols indicates no significant differences between contrasts.

Regarding bacterial samples in the absence of stress, higher values (*p*-value ≤ 0.1) were observed for Shannon and Gini-Simpson diversity indices in leaves than in roots of the control treatment (Figure 1A). For the vermicompost and millicompost HA treatments, it was observed that roots presented greater species richness than leaves; however, unlike what was observed in the treatment without HA, the application of HA did not affect the diversity indices between leaves and roots. The imposition of stress causes these differences to disappear (Figure 1A). In addition to the evaluations of the effect of the factors separately, it is notable that the combination of the factors treatment with humic acids and stress significantly influenced the Chao1 and Shannon indices (Supplementary Table S9).

Regarding the fungal composition, in relation to the difference between roots and leaves, greater species richness and diversity were observed in roots than in leaves in the absence of stress (Figure 1B). Similar behavior was observed in stressed plants, but only in the control treatment (Chao1 and Shannon). The differences that were observed in relation to the different tissues of unstressed plants seem to have been attenuated or extinguished in stressed plants, especially in those that received HA applications (Figure 1B). In the absence of stress, application of millicompost HA showed higher Chao1 values in roots compared to treatments with vermicompost HA and the control and higher values of the diversity indices compared to the control (Figure 1A). The application of HA did not significantly alter the alpha diversity metrics (Figure 1B). However, the interaction of the three factors (HA application + plant tissue + stress) had a significant effect on the diversity indices (Shannon and Gini-Simpson; Supplementary Table S10).

**Figure 2:**
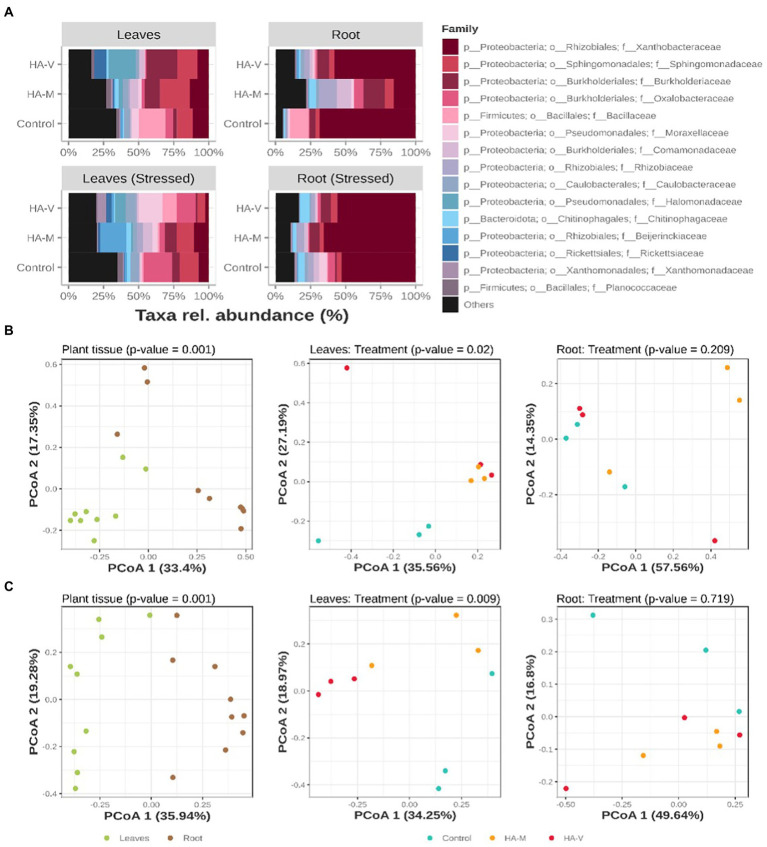
Relative abundances of the top 15 bacterial ASVs, collapsed to the taxonomic level of “family” **(A)**. Graphic representation of principal coordinate analysis (PCoA) based on the Bray–Curtis distances of samples in the absence **(B)** and presence **(C)** of stress. At the first level (on the left), the PCoAs of all samples tested in terms of separation by plant material are observed. Next (center and right), the distribution of leaf and root samples, respectively, tested according to the treatment received is observed. Treatments refer to the application of vermicompost HA (HA-V), millicompost HA (HA-M) and that did not receive application (control).

Considering the taxonomic level of the family, it was observed that the highest abundance of taxa was within the family *Proteobacteria,* and the other families, present in smaller proportions, were *Firmicutes* and *Bacteroidetes* (Figure 2A). Regarding roots, it was observed that the most abundant group was *Xanthobactereaceae*, and unstressed roots that received millicompost HA showed a greater plurality of families than roots of the control and those that received vermicompost HA; in addition, there was a greater abundance of *Rhizobiaceae*. This family comprises rhizobia, bacteria associated with BNF in soybean (Breedveld and Miller, 1994). In the presence of stress, in roots with millicompost HA, there was an increase in *Xanthobactereaceae* and a relative reduction in the abundance of the other groups.

Few studies have evaluated the effect of humic acids on microbial diversity in plants. Sun et al. (2022) verified that HA application in tomatoes resulted in the best flavor compared to the other two organic fertilizers. The Chaol estimator and Shannon index showed that fertilizer addition decreased microbial diversity but increased species richness. Akimbekov et al. (2020) demonstrated that HA increased potato growth (54.9%) and tuber yield (66.4%) when compared to the control and maintained the biogeochemical stability of soils, healthy microbial community structure, and increased the agronomic productivity of potato plants. Other studies have shown that the bacterial composition during vermicomposting was split between the families *Proteobacteria*, *Bacteroidetes*, *Actinobacteria*, *Firmicutes* and *Verrucimicrobia*. *Proteobacteria* were most abundant at the beginning of the process, while after the 14th day, their abundance decreased but remained significant (Gómez-Brandón et al., 2011, 2012).

Regarding the effect of fertilization on the soil microbial community, Wang et al. (2017) verified the effect of organic and inorganic fertilization on the soil microbial diversity during 10 years of fertilizer applications. The results of this study show that inorganic fertilization decreased the richness of bacteria and increased the richness of fungi. The application of mineral fertilizers increased the abundance of some oligotrophic bacteria, such as *Bacteroidetes* and *Acidobacteria*. On the other hand, the application of organic fertilizers increased the abundance of coprotrophic bacteria, such as *Proteobacteria* (Wang et al., 2017), which is a heterogeneous class that includes free-living, symbiotic, and gram-positive bacteria and some integral intracellular bacteria that generally have important metabolic capabilities, such as biological nitrogen fixation and carbon fixation (Hallez et al., 2017).

Principal coordinate analysis (PCoA) using Bray–Curtis distances was performed to determine the dissimilarity of bacterial communities between shoot and root samples in the different treatments. Regardless of HA application and the presence of stress, the bacterial compositions of the shoots and roots were different (*p* = 0.001; Figures 2B,C). Significance through PERMANOVA was only obtained in leaf samples, regardless of the “stress” factor (Figures 2B,C). In line with the PCoA graph, the hierarchical grouping shows the separation of the two groups (Supplementary Figure S4). Under nonstressful conditions, the variance in leaf communities and the PERMANOVA result showed a significant difference (*p* = 0.02) between treatments, where it was possible to observe the separation of the two groups, in which the community of the control treatment was more distant from the communities of leaves that received vermicompost and millicompost HA. In the presence of stress, a greater distance between samples treated with different HA was observed, forming three distinct groups, indicating the difference between bacterial communities. These results indicate that the enriched bacterial groups appear to play distinct roles in plants under water stress conditions.

**Figure 3:**
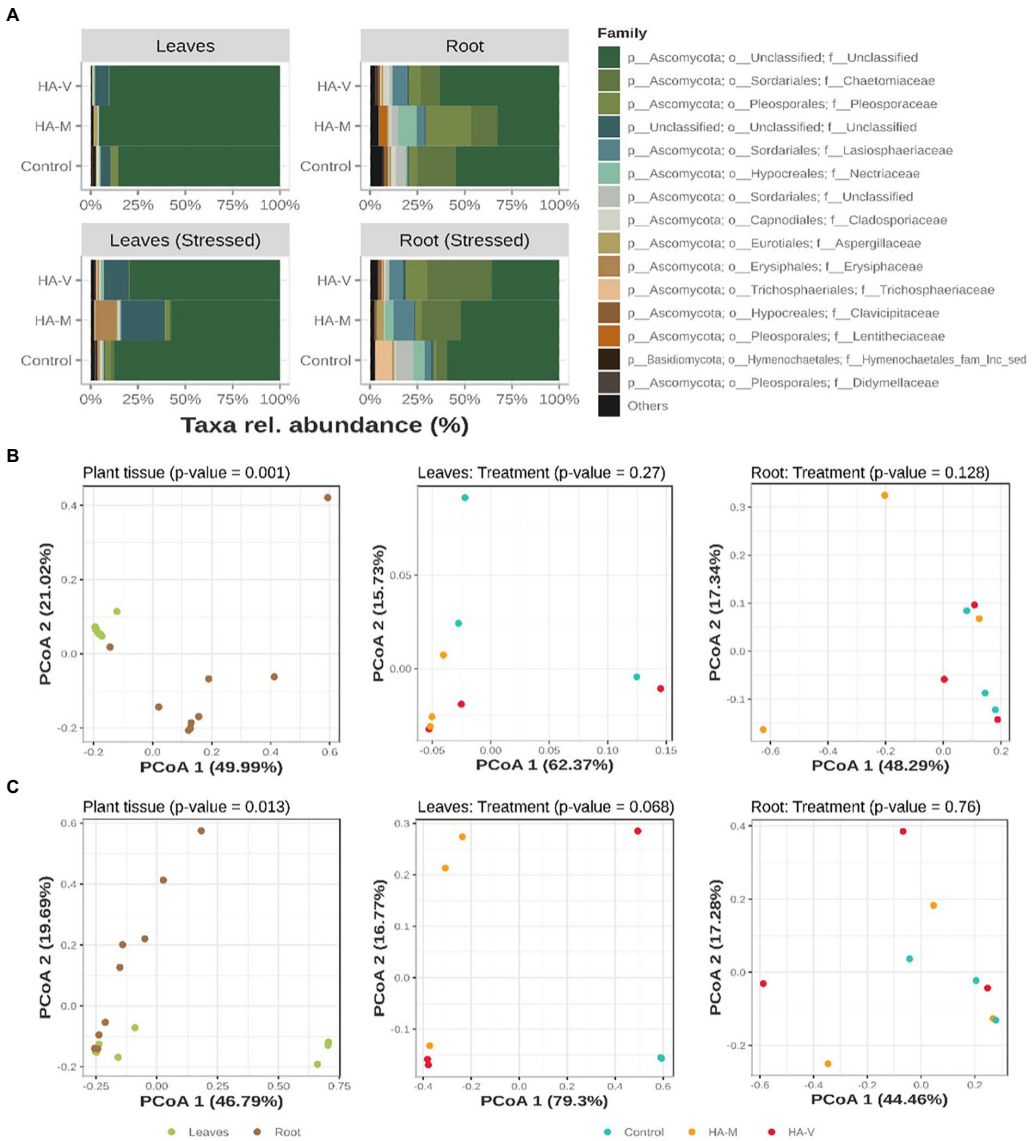
Relative abundances of the top 15 fungal ASVs, collapsed to the taxonomic level of “family” **(A)**. Graphic representation of principal coordinate analysis (PCoA) based on the Bray–Curtis distances of samples in the absence **(B)** and presence **(C)** of stress. At the first level (on the left), the PCoAs of all samples are observed and tested in terms of separation by plant material. Next (center and right), the distribution of leaf and root samples, respectively, tested according to the treatments received is observed. Treatments refer to the application of vermicompost HA (HA-V) and millicompost HA (HA-M) and that did not receive application (control).

For fungi, the taxa identified were mostly in the family *Ascomycota*, followed by *Basidiomycota* (Figures 3A). It is noteworthy that many of the most abundant sequences could not be classified even at the phylum level, as shown in Figure 3. Unstressed leaves presented mostly members related to the family *Ascomycota*. Specifically, in stressed leaves that received millicompost HA, an increase in the *Erysiphaceae* family was observed. In unstressed roots, the control showed an increase in members of the *Chaetomiaceae* family compared to the other treatments. Treatment with millicompost HA presented a greater abundance of *Nectriaceae* and *Pleosporaceae*. In the presence of stress, an increase in *Chaetomiaceae* was observed in treatments with HA, and an increase in *Trichosphaeriaceae* was observed in the control. For the distribution of compositions illustrated in PCoA (Figures 3B,C) and its respective dendrograms (Supplementary Figure S5), separation of leaf and root samples was observed (*p* < 0.05).

*Nectriaceae*, *Pleosporaceae* and *Trichosphaeriaceae* are families of fungi that are pathogenic to Berchemia discolor, which is a drought-tolerant multipurpose tree with the potential to provide medicine, food and other commodities to drylands in several countries (Susan et al., 2022). *Chaetomiaceae* species are a wealthy source of enzymes with diverse biotechnological and industrial applications, such as PMO (polysaccharide monooxygenase), L-methioninase, β-1,3-glucanase, laccase, dextranase, lipolytic, pectinolytic, amylolytic, chitinolytic, and proteolytic enzymes. Different classes of secondary metabolites have been reported from this family and are derived from various biosynthetic pathways, such as alkaloids, polyketides, peptides, terpenes, and polyketide-amino acid hybrid secondary metabolites. These metabolites have attracted research interest due to their fascinating structural frameworks and bioactivities (Ibrahim et al., 2021). The family *Erysiphe* (including powdery mildew fungi only known as anamorph, Pseudoidium) is the largest genus in *Erysiphaceae* and contains more than 50% of all species in this family. Little is known about the phylogenetic structure of this genus. Erysiphaceae (Ascomycete: Erysiphales) are a group of obligate parasitic fungi of plants that cause powdery mildew diseases on ~10,000 angiosperm species (Amano, 1986). With the exception of the dormant stage, their life cycle completely depends on living hosts, from which they obtain nutrients without killing the host cells and without which they are unable to survive. To maintain the obligate parasitic life cycle, *Erysiphaceae* have developed highly specific and sophisticated mechanisms to avoid the resistance system of the host, to obtain nutrient resources from the host without injuring the host cells, and to synchronize their life-cycle parameters to those of the host (Aist and Bushnell, 1991).

To evaluate the compositional differences among microbial communities present in the leaf and root samples, we detected differentially abundant (DA) taxa between control and HA-treated samples in the presence and absence of stress. The list of DA taxa, as well as for which condition and in what proportion they were identified, is available in more detail in Supplementary Tables S7 (Bacteria), S8 (Fungi).

The application of HA had a great effect on the bacterial communities. In the absence of stress, treated leaves tended to considerably reduce the abundance of certain taxa compared to the control (Table 3). In the other conditions, treatments with HA were superior in this regard, causing an increase in the abundance of specific taxa (Supplementary Tables S7, S8).

**Table 3:** Quantifications of differentially abundant (DA) taxa related to each treatment under the experimental conditions evaluated.

	Treatment	No stress		Stressed	
		Leaves	Roots	Leaves	Roots
Bacteria (16S rRNA)	HA-V	5	15	38	33
	HA-M	2	51	15	22
	Control	22	4	1	15
Fungi (ITS)	HA-V	3	9	–	27
	HA-M	3	26	–	11
	Control	2	3	–	9

HA-V, Samples treated with vermicompost HA; HA-M, Samples treated with millicompost HA. Control refers to samples without the addition of HA.

**Figure 4:**
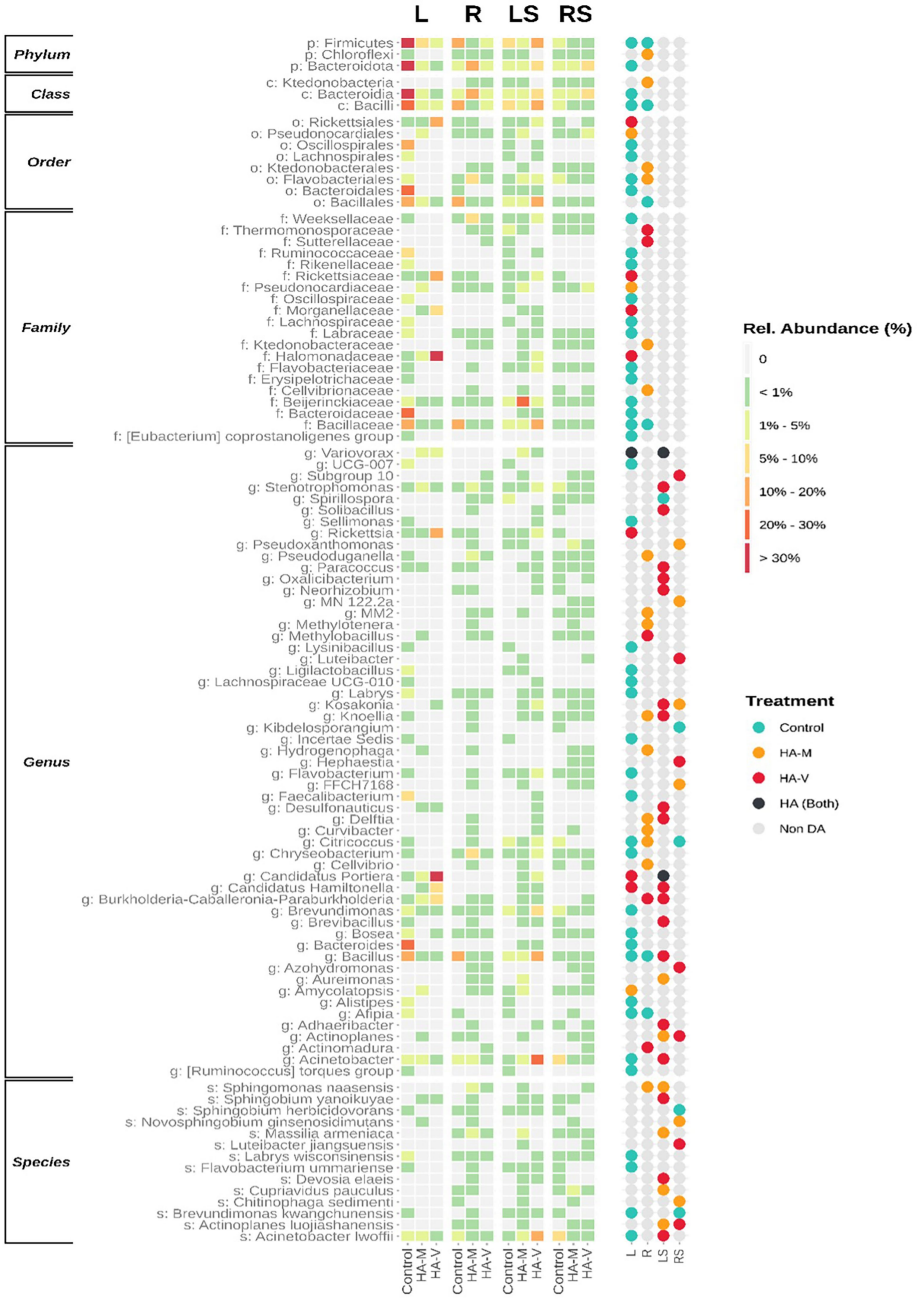
Differently abundant (DA) bacterial taxa. Both levels are presented ordered by the sum of relative abundances. The heatmap (left) demonstrates the relative abundance of taxa in leaves (L), roots (R), stressed leaves (LS), stressed roots (RS) by applying millicompost humic acid (HA-M), vermicompost humic acid (HA-V) and without application of HA (control). The dot plot (right) reveals in which niche differences in abundance between plant materials occurred.

In unstressed leaves, it was observed that the application of vermicompost HA promoted an increase in the genera *Rickettsia, Candidatus hamiltonella, and Candidatus portiera* (Figure 4), recognized endosymbionts of whitefly, a pest that causes damage to soybean plants (Bello et al., 2021). Despite the presence of these taxa, the prevalence of these groups was not observed in all replicates, possibly being a punctual contamination of one of the biological replicates. Despite this, the confirmation of these microbial groups shows that insect symbionts can enter plant tissues.

The genera *Variovorax* and *Kosakonia* were DA in plants that received HA. These bacterial groups have already been isolated from soybean plants and, when evaluated *in vitro*, showed antagonistic activity against various bacterial and fungal pathogens of this crop (De Almeida Lopes et al., 2018). Members of the genera *Variovorax* and *Oxalicibacterium* have been reported to catabolize oxalate (Tamer et al., 2002). In some plants, the degradation of this compound provides protection against fungal pathogens (Müller et al., 2016). Additionally, microorganisms that degrade oxalate can also act to increase nutrient uptake by plants (Morris and Allen, 1994).

A study evaluated the foliar application of leonardite (a compound with high HA concentrations) in beetroot produced under hydroponics and in field conditions and evaluated the effect of this compound on the endophytic bacterial community. The authors observed that the genus most responsive to the application of leonardite in beetroot under both conditions was *Oxalicibacterium* (Della Lucia et al., 2021). This genus was also stimulated in plants that received vermicompost HA. Therefore, the effects observed in the treatment with leonardite may be additional (or mediated) to the effects of bacterial endophytes.

In the treatment with millicompost HA, increases in *Cupriavidus pauculus, Sphingomonas naasensis, and Massilia armeniaca* were observed, which are genera that have members related to tolerance to heavy metals, growth under low water availability, resistance to microbial agents and assistance in nutrient uptake by plants (Menon et al., 2019; Asaf et al., 2020; Zeng et al., 2020). Therefore, these bacterial groups may act on plant growth and protect against abiotic stresses. *Actinoplane luojiashanensis* was also DA in this treatment, and one of the few studies found with this species highlights its characteristic in antibiotic production (Jilian et al., 1995). This genus, *Chitinophaga,* is commonly associated with biological control (Hu et al., 2019).

Some studies found *Novosphingobium ginsenosidimutans* to be highly associated with the soybean rhizosphere (Meier et al., 2021), and HA may have favored its endophytic colonization in this plant.

In plants treated with vermicompost HA, an increase in *Acinetobacter lwoffii* was observed, which has already been described for improving bean growth through arsenic contamination (Das and Sarkar, 2018). This same work observed the effect of this species on the production of plant regulators under favorable environmental conditions, such as under stress. Additionally, it has also been successful in improving the plant’s response to oxidative stress. *Sphingobium yanoikuyae* also plays a known role in protecting plants, providing tolerance to water stress (Arunthavasu et al., 2019). *Luteibacter jiangsuensis* is a bacterial species related to the degradation of complex molecules, including compounds toxic to the environment (Lin et al., 2020). Members of the genus *Devosia* have the ability to nodule legumes (Rivas et al., 2002) and may play a relevant role in this regard in relation to soybeans, since *Devosia elaeis* was DA in samples. *Actinoplane luojiashanensis* was DA in the vermicompost and millicompost HA treatments.

A study showed that the root application of HA was able to change the bacterial composition in this plant organ, providing an increase in taxa belonging to *Sphingobacteriaceae, Chitinophaga* and *Actinobacteria* (da Silva et al., 2021b). Similar results were found in the present work. The authors emphasize that the role of HA in inducing plant tolerance may be related to the stimulation of these bacteria. Therefore, it is appropriate to suggest that HA improves the physiological response of the plant to cope with environmental stresses (Canellas et al., 2020), while it also selects microorganisms with a role in promoting plant growth and protecting the plant from stress.

**Figure 5:**
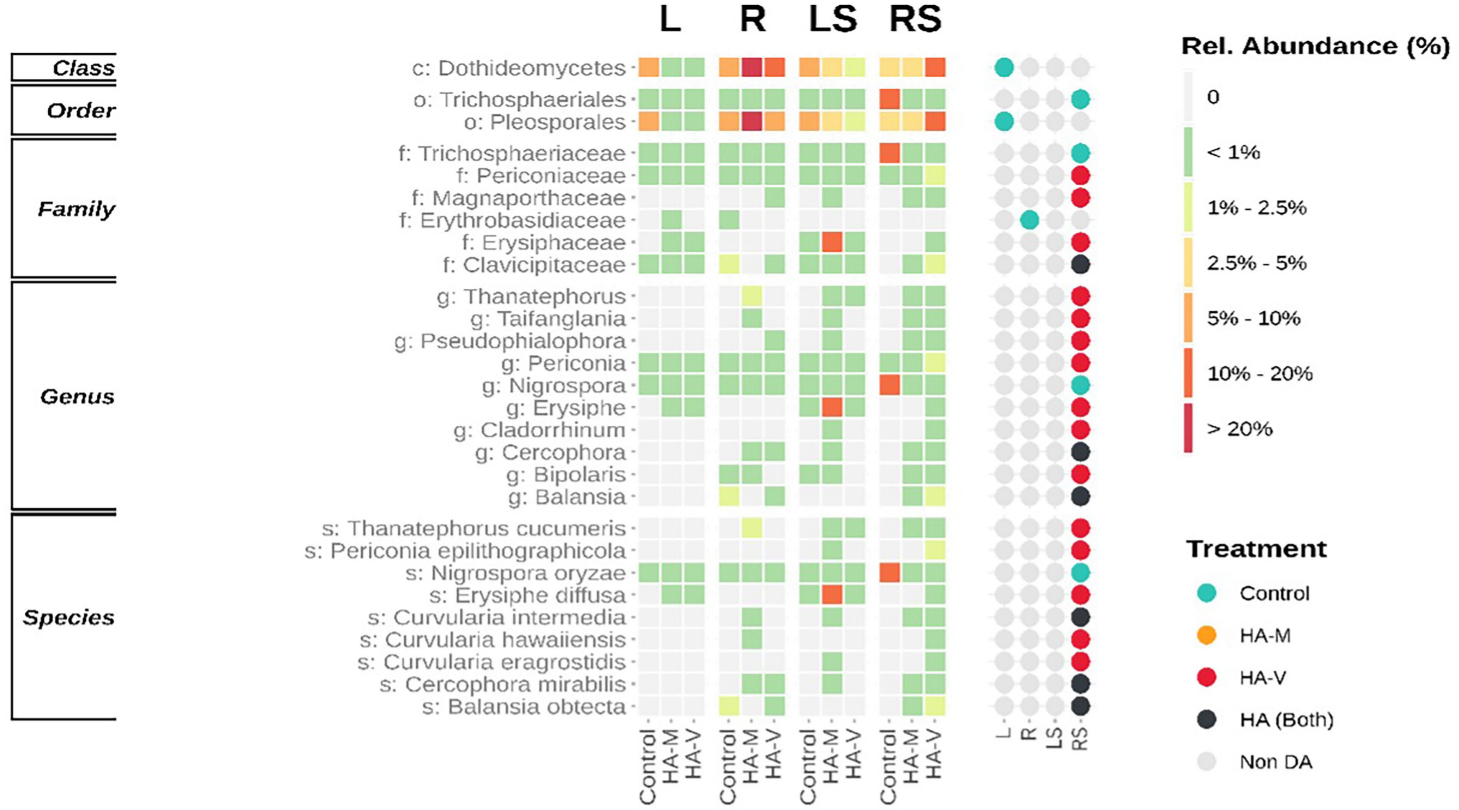
Different abundant (DA) fungal taxa. Both levels are presented ordered by the sum of relative abundances. The heatmap (left) demonstrates the relative abundance of taxa in leaves (L), roots (R), stressed leaves (LS), stressed roots (RS) by applying millicompost humic acid (HA-M), vermicompost humic acid (HA-V) and without application of HA (control). The dot plot (right) reveals in which niche differences in abundance between plant materials occurred.

Regarding the fungal portion, DA groups were observed in stressed roots, where treatments with HA were more impactful, especially treatment with vermicompost HA. At the species level, treatments with HA reduced *Nigrospora oryzae* (Figure 5). Interestingly, some enriched fungal groups are related to phytopathogens, such as *Erysiphe diffusa* (McTaggart et al., 2012) and *Thanatephorus cucumeris* (Chela Fenille et al., 2002). Despite this, no disease was observed in soybean plants. It is possible that different responses to plant growth promotion and pathogenicity are related to changes in the genotype of the plant and microorganisms. Such changes can generate changes in recognition and the type of interaction between them (Schmidt et al., 2012). Furthermore, the microbiome itself can affect interactions between microorganisms and plants.

The species *Periconia epilithographicola* was enriched in the presence of HA, and its genus has members with a role in biocontrol (Verma et al., 2011). *Cercophora mirabilis*, a species of this genus, has been found to be associated with soybean roots in the field, but its function is still poorly understood (Bargaz et al., 2017). The genus *Balansia* has been reported as a pathogen, growth promoter and antagonist of agricultural pests (Clay et al., 1985; Porter et al., 1985; Ren and Clay, 2009). *Curvularia* also plays a dubious role in its effect on the plant, since this genus presents individuals who act in plant growth promotion and are capable of causing diseases in plants (Michael Tilley and Lynn Walker, 2002).

It is known that under abiotic stress, opportunistic pathogens more easily colonize previously weakened plants, reducing their defense against injuries (Tewari and Arora, 2014). Thus, the stimulation of microorganisms related to biological control is also of great relevance for plant establishment even under abiotic stresses. In nature, most plants are colonized by an enormous diversity of endophytic and pathogenic microorganisms. However, it is still unclear how the presence of multiple partners is balanced in plant roots to the point of maintaining plant growth (Niu et al., 2020).

In short, the application of millicompost and vermicompost HA helps plant growth and induces the modulation of the endophytic community. It was also shown that HA can trigger the enrichment of microorganisms with the potential to act both in plant growth and defense against pathogens and protection against abiotic stresses. These results support previous questions, such as the need for approaches to elucidate the real role of HA in plant physiology and the participation of plant microbiota. Our findings may point to the use of HA as part of a strategy to prepare plants against biotic and abiotic stresses by stimulating their defense metabolism.

It is noteworthy that perhaps the application of HA has favored the endophytic establishment of soil microorganisms, including opportunistic fungi. However, it is suggested that the organization of the microbiota is adjusted to prevent the development of diseases through these potentially pathogenic groups. Thus, the present study represents an advance in the understanding of HA-induced changes in the soybean microbiome and reinforces the potential of this compound to improve plant growth even under water restrictions. However, more studies should be developed to understand the effect of HA on the plant microbiota and its function in plants.

## Conclusion

HA extracted from vermicompost and millicompost has the potential to promote the maintenance of plant growth even in adverse environmental conditions, acting in the best performance of the plant in the face of environmental stresses, as well as recruiting beneficial microorganisms to the plant. Although the results showed slight differences between vermicompost and millicompost regarding plant growth, both humic acids promoted an increase in plant development compared to the control. These substances could be used due to their beneficial function in the plant and its microbiome. HA can behave as a sustainable agricultural technology, promoting plant growth and thus reducing the need for agrochemicals.

## Data availability statement

The datasets presented in this study can be found in online repositories. The names of the repository/repositories and accession number(s) can be found in the article/supplementary material.

## Author contributions

All authors listed have made a substantial, direct, and intellectual contribution to the work and approved it for publication.

## Funding

This study was financed in part by the Coordination for the Improvement of Higher Education Personnel (CAPES), Brazil, Finance Code 001.

## Conflict of interest

The authors declare that the research was conducted in the absence of any commercial or financial relationships that could be construed as a potential conflict of interest.

## Publisher’s note

All claims expressed in this article are solely those of the authors and do not necessarily represent those of their affiliated organizations, or those of the publisher, the editors and the reviewers. Any product that may be evaluated in this article, or claim that may be made by its manufacturer, is not guaranteed or endorsed by the publisher.
