# Supplementary material for: Effect of the application of vermicompost and millicompost humic acids about the soybean microbiome under water restriction conditions
Source: Front Microbiol. 2022 Nov 4;13:1000222. doi: 10.3389/fmicb.2022.1000222 (PMC9672817; doi:10.3389/fmicb.2022.1000222)
Supplement: Supplementary file 1 [file Data_Sheet_1.docx]

**Effect of the application of vermicompost and millicompost humic acids on the soybean microbiome under water restriction conditions**

Maura Santos Reis de Andrade da Silva^a,c^, Lucas Amoroso Lopes de Carvalho^a^, Lucas Boscov Braos^b^, Luiz Fernando de Sousa Antunes^c^, Camilla Santos Reis de Andrade da Silva^d^, Cleudison Gabriel Nascimento da Silva^e^, Daniel Guariz Pinheiro^a^, Maria Elizabeth Fernandes Correia^f^, Ednaldo da Silva Araújo^f^, Luiz Alberto Colnago^g^, Nicolas Desoignies^h^, Everaldo Zonta^d^, Everlon Cid Rigobelo^a*^.

^a^Programa de Pós-graduação em Microbiologia Agropecuária, Universidade Estadual Paulista (UNESP), Faculdade de Ciências Agrárias e Veterinárias, Jaboticabal, SP, Brazil

^b^Programa de Pós-graduação em Agronomia, Universidade Estadual Paulista (UNESP), Faculdade de Ciências Agrárias e Veterinárias, Jaboticabal, SP, Brazil

^c^Universidade Federal Rural do Rio de Janeiro (UFRRJ), Seropédica, RJ, Brazil

^d^Departamento de Solos - Instituto de Agronomia - Universidade Federal Rural do Rio de Janeiro (UFRRJ), Seropédica, RJ, Brazil.

^e^Programa de Pós-graduação em Microbiologia Agrícola, Universidade Federal de Lavras (UFLA), Lavras, MG, Brazil

^f^National Agrobiology Research Center, Embrapa Agrobiologia, Seropédica, RJ, Brazil

^g^National Agrobiology Research Center, Embrapa Instrumentation, São Carlos, SP, Brazil

^h^Phytopathology, Microbial and Molecular Farming Lab, Centre D'Etudes et Recherche Appliquée-Haute Ecole Provinciale du Hainaut Condorcet, Ath, Belgium.

**Supplementary Material**

| **Table S1.** Counts of 16S rRNA amplicon reads throughout data processing, per sample. The table comprises the initial postsequencing quantification (Raw) and the steps of quality control and base pruning and low-quality sequences (QC and Trimming), merged sequence pairs (Merged), classified as Amplicon Variant Sequences (ASVs), and free of host contaminants (mitochondria and chloroplasts; Usable ASVs). |
| --- |
| \| **Sample** \| **Raw** \| **QC and Trimming** \| **Merged** \| **ASV** \| **Usable ASV** \| \| --- \| --- \| --- \| --- \| --- \| --- \| \| L_C_N_1 \| 93,729 \| 92,872 \| 92,553 \| 91,300 \| 18,992 \| \| L_C_N_2 \| 90,912 \| 90,052 \| 89,692 \| 88,041 \| 2,034 \| \| L_C_N_3 \| 92,503 \| 91,711 \| 91,309 \| 89,107 \| 2,158 \| \| L_C_S_1 \| 80,115 \| 79,222 \| 78,954 \| 77,557 \| 7,537 \| \| L_C_S_2 \| 54,740 \| 53,098 \| 52,808 \| 51,422 \| 2,827 \| \| L_C_S_3 \| 91,353 \| 90,375 \| 90,035 \| 84,293 \| 11,352 \| \| L_HM_N_1 \| 105,213 \| 104,106 \| 103,678 \| 99,819 \| 3,218 \| \| L_HM_N_2 \| 87,282 \| 86,219 \| 85,847 \| 84,286 \| 1,392 \| \| L_HM_N_3 \| 88,689 \| 87,896 \| 87,533 \| 85,739 \| 1,518 \| \| L_HM_S_1 \| 97,618 \| 96,519 \| 96,166 \| 94,276 \| 1,635 \| \| L_HM_S_2 \| 98,709 \| 97,702 \| 97,319 \| 95,388 \| 8,223 \| \| L_HM_S_3 \| 92,353 \| 91,526 \| 91,187 \| 89,474 \| 8,939 \| \| L_HV_N_1 \| 90,631 \| 89,856 \| 89,470 \| 87,985 \| 1,923 \| \| L_HV_N_2 \| 91,172 \| 90,409 \| 90,074 \| 88,469 \| 17,304 \| \| L_HV_N_3 \| 85,401 \| 84,683 \| 84,344 \| 82,734 \| 1,330 \| \| L_HV_S_1 \| 84,933 \| 84,132 \| 83,965 \| 82,922 \| 56,869 \| \| L_HV_S_2 \| 86,911 \| 86,052 \| 85,773 \| 83,945 \| 19,561 \| \| L_HV_S_3 \| 95,541 \| 94,682 \| 94,350 \| 92,956 \| 19,179 \| \| R_C_N_1 \| 81,082 \| 80,332 \| 80,004 \| 78,372 \| 24,900 \| \| R_C_N_2 \| 97,779 \| 96,852 \| 96,508 \| 95,377 \| 44,683 \| \| R_C_N_3 \| 82,913 \| 82,131 \| 81,834 \| 80,773 \| 41,441 \| \| R_C_S_1 \| 82,360 \| 81,616 \| 81,281 \| 79,442 \| 10,462 \| \| R_C_S_2 \| 77,180 \| 76,427 \| 76,221 \| 75,175 \| 50,523 \| \| R_C_S_3 \| 78,380 \| 77,614 \| 77,426 \| 76,711 \| 58,036 \| \| R_HM_N_1 \| 85,078 \| 84,280 \| 84,093 \| 82,400 \| 51,524 \| \| R_HM_N_2 \| 77,546 \| 76,780 \| 76,643 \| 75,504 \| 55,641 \| \| R_HM_N_3 \| 78,885 \| 78,136 \| 77,853 \| 76,226 \| 27,462 \| \| R_HM_S_1 \| 80,724 \| 79,964 \| 79,761 \| 79,028 \| 59,397 \| \| R_HM_S_2 \| 78,179 \| 77,312 \| 77,139 \| 75,719 \| 51,072 \| \| R_HM_S_3 \| 90,440 \| 89,503 \| 89,176 \| 87,019 \| 23,771 \| \| R_HV_N_1 \| 82,830 \| 82,172 \| 81,846 \| 80,633 \| 16,445 \| \| R_HV_N_2 \| 90,916 \| 90,029 \| 89,752 \| 88,503 \| 46,073 \| \| R_HV_N_3 \| 90,823 \| 89,945 \| 89,498 \| 87,264 \| 3,412 \| \| R_HV_S_1 \| 76,332 \| 75,685 \| 75,475 \| 74,802 \| 53,641 \| \| R_HV_S_2 \| 75,619 \| 74,813 \| 74,644 \| 73,231 \| 55,546 \| \| R_HV_S_3 \| 91,584 \| 90,641 \| 90,251 \| 88,310 \| 25,512 \| |
| The sample names represent the combination of factors: Plant tissue (Leaf [L] or Root [R]), type of humic acid (vermicompost [HV], millicompost [HM] or control [C]), and condition of water stress (unstressed [N] or stressed [S]). |
| **Table S2.** Counts of ITS amplicon reads throughout data processing, per sample. The table comprises the initial postsequencing quantification (Raw) and the steps of quality control and base pruning and low-quality sequences (QC and Trimming), merged sequence pairs (Merged), classified as Amplicon Variant Sequences (ASVs), and free of host contaminants (mitochondria and chloroplasts; Usable ASVs). |
| \| **Sample** \| **Raw** \| **QC and Trimming** \| **Merged** \| **ASV** \| **Usable ASV** \| \| --- \| --- \| --- \| --- \| --- \| --- \| \| L_C_N_1 \| 45,640 \| 43,506 \| 43,222 \| 42,827 \| 42,232 \| \| L_C_N_2 \| 66,166 \| 64,745 \| 64,600 \| 64,033 \| 63,871 \| \| L_C_N_3 \| 51,065 \| 49,743 \| 49,609 \| 49,211 \| 48,761 \| \| L_C_S_1 \| 15,083 \| 13,722 \| 13,611 \| 13,446 \| 12,478 \| \| L_C_S_2 \| 55,514 \| 52,989 \| 52,522 \| 52,152 \| 50,906 \| \| L_HM_N_1 \| 66,202 \| 64,179 \| 63,898 \| 63,281 \| 62,713 \| \| L_HM_N_2 \| 67,092 \| 65,463 \| 65,240 \| 64,681 \| 64,151 \| \| L_HM_N_3 \| 68,155 \| 66,581 \| 66,336 \| 65,685 \| 65,451 \| \| L_HM_S_1 \| 74,361 \| 72,698 \| 72,395 \| 71,612 \| 71,252 \| \| L_HM_S_2 \| 68,554 \| 67,124 \| 66,665 \| 66,058 \| 65,783 \| \| L_HM_S_3 \| 61,937 \| 60,823 \| 60,574 \| 60,073 \| 58,995 \| \| L_HV_N_1 \| 50,218 \| 48,613 \| 48,436 \| 47,957 \| 47,620 \| \| L_HV_N_2 \| 49,808 \| 48,541 \| 40,029 \| 39,650 \| 38,932 \| \| L_HV_N_3 \| 62,094 \| 60,733 \| 60,471 \| 59,901 \| 58,894 \| \| L_HV_S_1 \| 81,574 \| 77,940 \| 77,259 \| 76,815 \| 73,536 \| \| L_HV_S_2 \| 28,781 \| 27,780 \| 26,987 \| 26,662 \| 26,382 \| \| L_HV_S_3 \| 76,452 \| 74,290 \| 73,911 \| 73,223 \| 72,726 \| \| R_C_N_1 \| 76,433 \| 70,139 \| 68,989 \| 68,563 \| 66,409 \| \| R_C_N_2 \| 69,407 \| 66,352 \| 65,619 \| 65,131 \| 59,235 \| \| R_C_N_3 \| 62,697 \| 60,901 \| 60,065 \| 59,350 \| 56,864 \| \| R_C_S_1 \| 48,132 \| 47,070 \| 46,449 \| 46,011 \| 45,672 \| \| R_C_S_2 \| 62,422 \| 60,894 \| 60,656 \| 60,043 \| 58,493 \| \| R_C_S_3 \| 72,978 \| 71,386 \| 71,168 \| 70,426 \| 68,645 \| \| R_HM_N_1 \| 68,159 \| 66,894 \| 63,489 \| 62,963 \| 53,657 \| \| R_HM_N_2 \| 80,597 \| 78,799 \| 76,983 \| 76,558 \| 41,808 \| \| R_HM_N_3 \| 78,691 \| 76,483 \| 75,340 \| 74,540 \| 70,035 \| \| R_HM_S_1 \| 78,739 \| 77,341 \| 76,731 \| 76,219 \| 75,376 \| \| R_HM_S_2 \| 62,673 \| 60,428 \| 59,394 \| 58,948 \| 49,729 \| \| R_HM_S_3 \| 59,403 \| 57,466 \| 57,073 \| 56,425 \| 55,406 \| \| R_HV_N_1 \| 81,710 \| 79,709 \| 79,196 \| 78,570 \| 76,950 \| \| R_HV_N_2 \| 50,263 \| 49,139 \| 48,049 \| 47,426 \| 46,241 \| \| R_HV_N_3 \| 59,346 \| 57,120 \| 56,453 \| 55,803 \| 53,995 \| \| R_HV_S_1 \| 71,382 \| 68,978 \| 67,707 \| 67,205 \| 65,560 \| \| R_HV_S_2 \| 75,855 \| 74,804 \| 74,380 \| 74,068 \| 73,354 \| \| R_HV_S_3 \| 71,887 \| 70,098 \| 68,946 \| 68,412 \| 68,015 \| |
| The sample names represent the combination of factors: Plant tissue (Leaf [L] or Root [R]), type of humic acid (vermicompost [HV], millicompost [HM] or control [C]), and condition of water stress (unstressed [N] or stressed [S]). |

| **Table S3.** Counts of 16S rRNA amplicon reads throughout data processing, per group. The table comprises the initial postsequencing quantification (Raw) and the steps of quality control and base pruning and low-quality sequences (QC and Trimming), merged sequence pairs (Merged), classified as Amplicon Variant Sequences (ASVs), and free of host contaminants (mitochondria and chloroplasts; Usable ASVs). |
| --- |
| \| **Sample** \| **Raw** \| **QC and Trimming** \| **Merged** \| **ASV** \| **Usable ASV** \| \| --- \| --- \| --- \| --- \| --- \| --- \| \| L_C_N \| 277,144 \| 274,635 \| 273,554 \| 268,448 \| 23,184 \| \| L_C_S \| 226,208 \| 222,695 \| 221,797 \| 213,272 \| 21,716 \| \| L_HM_N \| 281,184 \| 278,221 \| 277,058 \| 269,844 \| 6,128 \| \| L_HM_S \| 288,680 \| 285,747 \| 284,672 \| 279,138 \| 18,797 \| \| L_HV_N \| 267,204 \| 264,948 \| 263,888 \| 259,188 \| 20,557 \| \| L_HV_S \| 267,385 \| 264,866 \| 264,088 \| 259,823 \| 95,609 \| \| R_C_N \| 261,774 \| 259,315 \| 258,346 \| 254,522 \| 111,024 \| \| R_C_S \| 237,920 \| 235,657 \| 234,928 \| 231,328 \| 119,021 \| \| R_HM_N \| 241,509 \| 239,196 \| 238,589 \| 234,130 \| 134,627 \| \| R_HM_S \| 249,343 \| 246,779 \| 246,076 \| 241,766 \| 134,240 \| \| R_HV_N \| 264,569 \| 262,146 \| 261,096 \| 256,400 \| 65,930 \| \| R_HV_S \| 243,535 \| 241,139 \| 240,370 \| 236,343 \| 134,699 \| |
| The sample names represent the combination of factors: Plant tissue (Leaf [L] or Root [R]), type of humic acid (vermicompost [HV], millicompost [HM] or control [C]), and condition of water stress (unstressed [N] or stressed [S]). |

| **Table S4.** Counts of ITS amplicon reads throughout data processing, per group. The table comprises the initial postsequencing quantification (Raw) and the steps of quality control and base pruning and low-quality sequences (QC and Trimming), merged sequence pairs (Merged), classified as Amplicon Variant Sequences (ASVs), and free of host contaminants (mitochondria and chloroplasts; Usable ASVs). |
| --- |
| \| **Sample** \| **Raw** \| **QC and Trimming** \| **Merged** \| **ASV** \| **Usable ASV** \| \| --- \| --- \| --- \| --- \| --- \| --- \| \| L_C_N \| 162,871 \| 157,994 \| 157,431 \| 156,071 \| 154,864 \| \| L_C_S \| 70,597 \| 66,711 \| 66,133 \| 65,598 \| 63,384 \| \| L_HM_N \| 201,449 \| 196,223 \| 195,474 \| 193,647 \| 192,315 \| \| L_HM_S \| 204,852 \| 200,645 \| 199,634 \| 197,743 \| 196,030 \| \| L_HV_N \| 162,120 \| 157,887 \| 148,936 \| 147,508 \| 145,446 \| \| L_HV_S \| 186,807 \| 180,010 \| 178,157 \| 176,700 \| 172,644 \| \| R_C_N \| 208,537 \| 197,392 \| 194,673 \| 193,044 \| 182,508 \| \| R_C_S \| 183,532 \| 179,350 \| 178,273 \| 176,480 \| 172,810 \| \| R_HM_N \| 227,447 \| 222,176 \| 215,812 \| 214,061 \| 165,500 \| \| R_HM_S \| 200,815 \| 195,235 \| 193,198 \| 191,592 \| 180,511 \| \| R_HV_N \| 191,319 \| 185,968 \| 183,698 \| 181,799 \| 177,186 \| \| R_HV_S \| 219,124 \| 213,880 \| 211,033 \| 209,685 \| 206,929 \| |
| The sample names represent the combination of factors: Plant tissue (Leaf [L] or Root [R]), type of humic acid (vermicompost [HV], millicompost [HM] or control [C]), and condition of water stress (unstressed [N] or stressed [S]). |

| **Table S5.** Counts of 16S rRNA amplicon reads classified in each taxonomic rank. The percentages in parentheses indicate the proportion of usable reads (free of contaminants) that could be classified up to a certain taxonomic rank. |
| --- |
| \| **Sample** \| **Phylum** \| **Class** \| **Order** \| **Family** \| **Genus** \| **Species** \| \| --- \| --- \| --- \| --- \| --- \| --- \| --- \| \| L_C_N \| 23,184 (100.00) \| 23,184 (100.00) \| 23,119 (99.72) \| 22,819 (98.43) \| 21,699 (93.59) \| 1,453 (6.27) \| \| L_C_S \| 21,716 (100.00) \| 21,606 (99.49) \| 21,299 (98.08) \| 20,568 (94.71) \| 18,769 (86.43) \| 680  (3.13) \| \| L_HM_N \| 6,128 (100.00) \| 6,109 (99.69) \| 6,001 (97.93) \| 5,571 (90.91) \| 5,029 (82.07) \| 261  (4.26) \| \| L_HM_S \| 18,797 (100.00) \| 18,773 (99.87) \| 18,671 (99.33) \| 18,394 (97.86) \| 16,941 (90.13) \| 2,056 (10.94) \| \| L_HV_N \| 20,557 (100.00) \| 20,543 (99.93) \| 20,461 (99.53) \| 20,196 (98.24) \| 19,915 (96.88) \| 136  (0.66) \| \| L_HV_S \| 95,609 (100.00) \| 95,551 (99.94) \| 95,384 (99.76) \| 94,989 (99.35) \| 93,538 (97.83) \| 21,560 (22.55) \| \| R_C_N \| 111,024 (100.00) \| 110,933 (99.92) \| 110,885 (99.87) \| 110,456 (99.49) \| 105,715 (95.22) \| 4,189 (3.77) \| \| R_C_S \| 119,021 (100.00) \| 119,017 (100.00) \| 118,858 (99.86) \| 118,609 (99.65) \| 115,524 (97.06) \| 9,275 (7.79) \| \| R_HM_N \| 134,627 (100.00) \| 134,594 (99.98) \| 134,126 (99.63) \| 132,914 (98.73) \| 119,512 (88.77) \| 13,310 (9.89) \| \| R_HM_S \| 134,240 (100.00) \| 134,237 (100.00) \| 134,199 (99.97) \| 132,944 (99.03) \| 125,133 (93.22) \| 5,473 (4.08) \| \| R_HV_N \| 65,930 (100.00) \| 65,930 (100.00) \| 65,897 (99.95) \| 65,599 (99.50) \| 63,642 (96.53) \| 1,384 (2.10) \| \| R_HV_S \| 134,699 (100.00) \| 134,670 (99.98) \| 134,565 (99.90) \| 133,957 (99.45) \| 123,243 (91.50) \| 5,118 (3.80) \| |
| The sample names represent the combination of factors: Plant tissue (Leaf [L] or Root [R]), type of humic acid (vermicompost [HV], millicompost [HM] or control [C]), and condition of water stress (unstressed [N] or stressed [S]). |

| **Table S6.** Counts of ITS amplicon reads classified in each taxonomic rank. The percentages in parentheses indicate the proportion of usable reads (free of contaminants) that could be classified up to a certain taxonomic rank. |
| --- |
| \| **Sample** \| **Phylum** \| **Class** \| **Order** \| **Family** \| **Genus** \| **Species** \| \| --- \| --- \| --- \| --- \| --- \| --- \| --- \| \| L_C_N \| 147,025 (94.94) \| 14,655 (9.46) \| 14,642 (9.45) \| 14,515 (9.37) \| 14,335 (9.26) \| 6,778 (4.38) \| \| L_C_S \| 63,048 (99.47) \| 7,677 (12.11) \| 7,639 (12.05) \| 7,579 (11.96) \| 7,011 (11.06) \| 5,308 (8.37) \| \| L_HM_N \| 191,780 (99.72) \| 7,676 (3.99) \| 7,663 (3.98) \| 7,542 (3.92) \| 7,477 (3.89) \| 7,006 (3.64) \| \| L_HM_S \| 152,906 (78.00) \| 39,525 (20.16) \| 39,431 (20.11) \| 38,644 (19.71) \| 37,841 (19.30) \| 35,908 (18.32) \| \| L_HV_N \| 132,993 (91.44) \| 3,109 (2.14) \| 3,061 (2.10) \| 3,043 (2.09) \| 3,030 (2.08) \| 2,742 (1.89) \| \| L_HV_S \| 143,687 (83.23) \| 11,201 (6.49) \| 11,167 (6.47) \| 10,957 (6.35) \| 10,561 (6.12) \| 9,572 (5.54) \| \| R_C_N \| 181,500 (99.45) \| 81,974 (44.92) \| 81,562 (44.69) \| 71,182 (39.00) \| 65,736 (36.02) \| 61,142 (33.50) \| \| R_C_S \| 170,967 (98.93) \| 66,709 (38.60) \| 66,652 (38.57) \| 53,598 (31.02) \| 53,224 (30.80) \| 49,458 (28.62) \| \| R_HM_N \| 164,152 (99.19) \| 106,327 (64.25) \| 106,282 (64.22) \| 99,292 (60.00) \| 96,002 (58.01) \| 84,002 (50.76) \| \| R_HM_S \| 179,986 (99.71) \| 91,661 (50.78) \| 91,624 (50.76) \| 90,155 (49.94) \| 87,743 (48.61) \| 84,979 (47.08) \| \| R_HV_N \| 175,694 (99.16) \| 67,038 (37.83) \| 66,739 (37.67) \| 64,562 (36.44) \| 62,360 (35.19) \| 50,260 (28.37) \| \| R_HV_S \| 204,199 (98.68) \| 132,396 (63.98) \| 131,633 (63.61) \| 128,036 (61.87) \| 121,132 (58.54) \| 113,604 (54.90) \| |
| The sample names represent the combination of factors: Plant tissue (Leaf [L] or Root [R]), type of humic acid (vermicompost [HV], millicompost [HM] or control [C]), and condition of water stress (unstressed [N] or stressed [S]). |

**
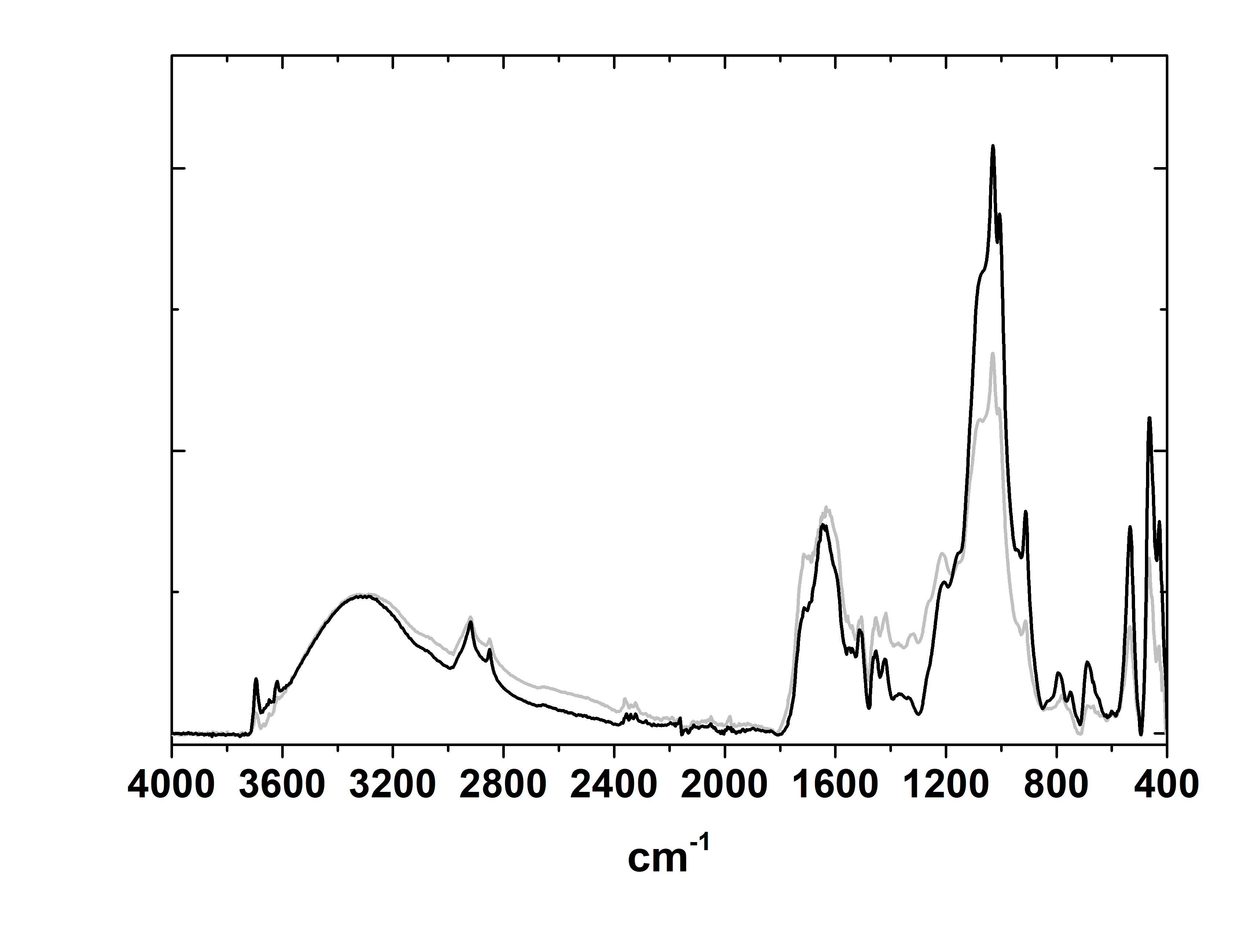
**

**Fig. S 1.** Infrared spectra of humic acid samples. In gray, the spectrum of millicompost HA and in black, vermicompost HA.

| **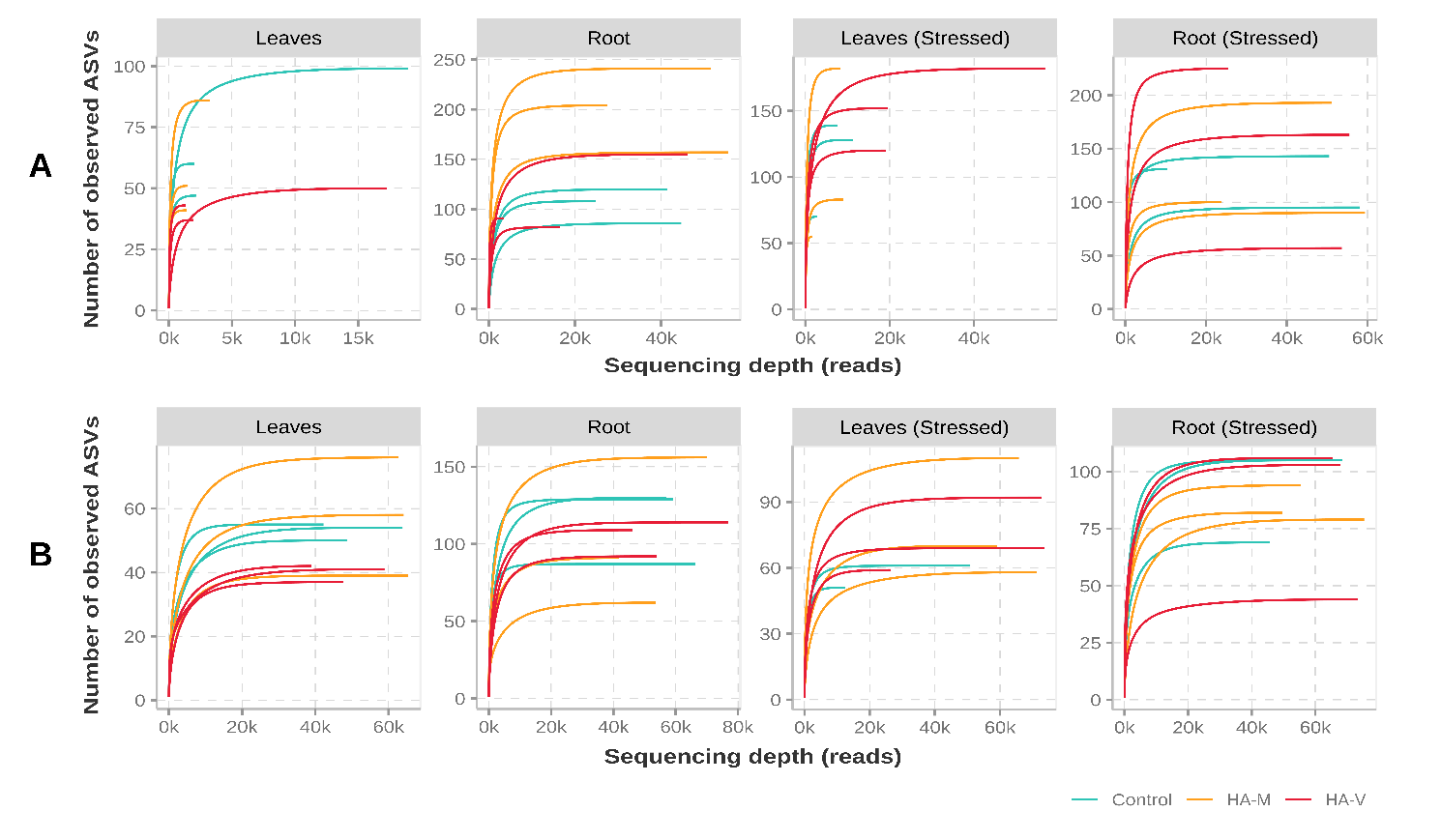** |
| --- |
| **Fig. S 2.** Rarefaction curve of bacterial (A) and fungal (B) ASVs in relation to sequencing depth. |

|  |
| --- |


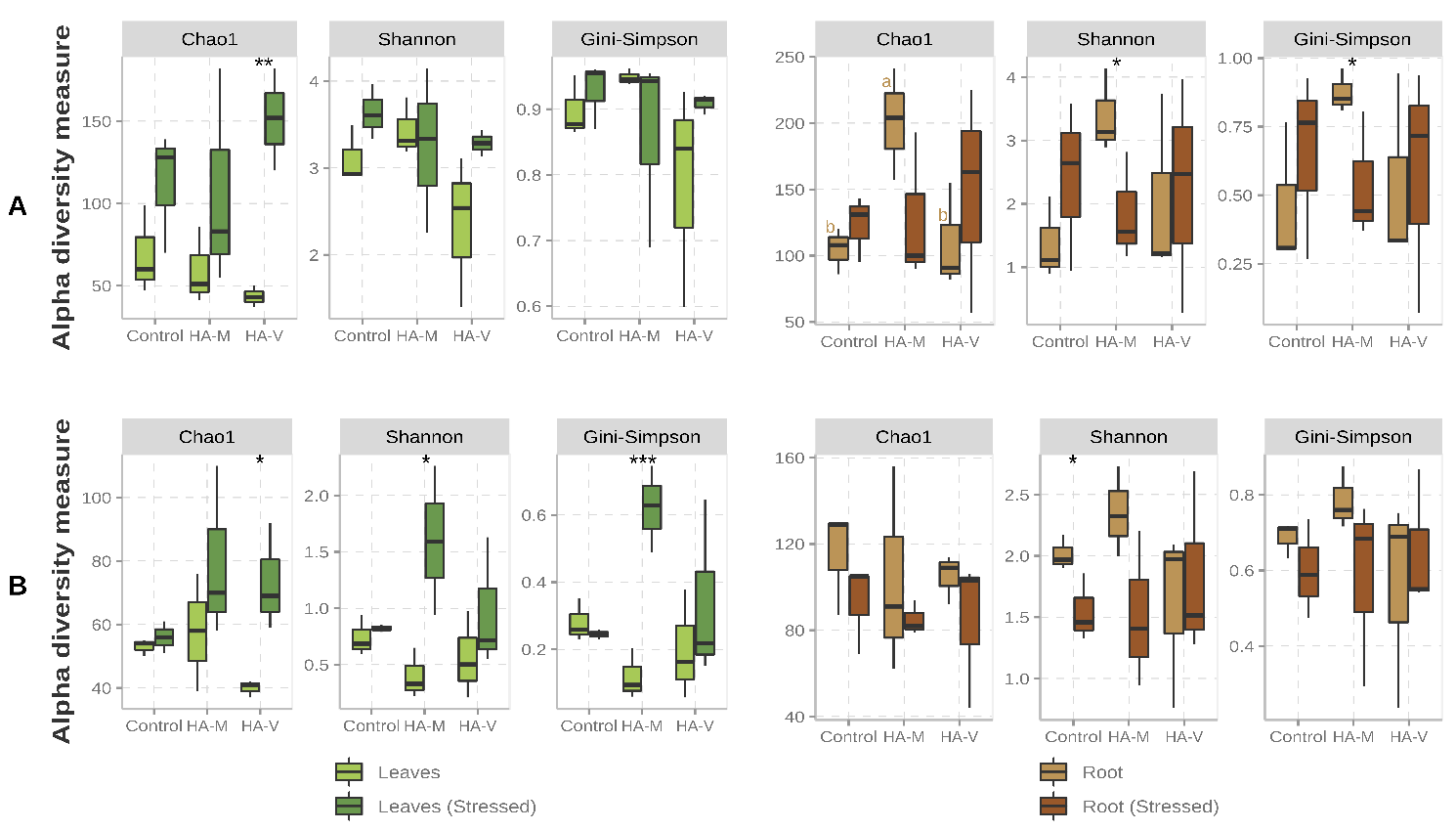


**Fig. S 3.** Boxplot of the alpha diversity metrics of bacterial (A) and fungal (B) fractions present in the shoot and root of soybean plants that received the application of vermicompost HA (HA-V) and millicompost HA (HA-M) or that did not receive application (Control) in the presence and absence of stress. Different letters indicate significant differences between HA application within the same plant material (*post hoc* test). Significant differences between different plant tissues as a function of stress can be seen by the presence of the following symbols: **** (p < 0.001); *** (p ≤ 0.01); ** (p ≤ 0.05); * (p ≤ 0.1). The absence of letters/symbols indicates no significant differences between contrasts.

| 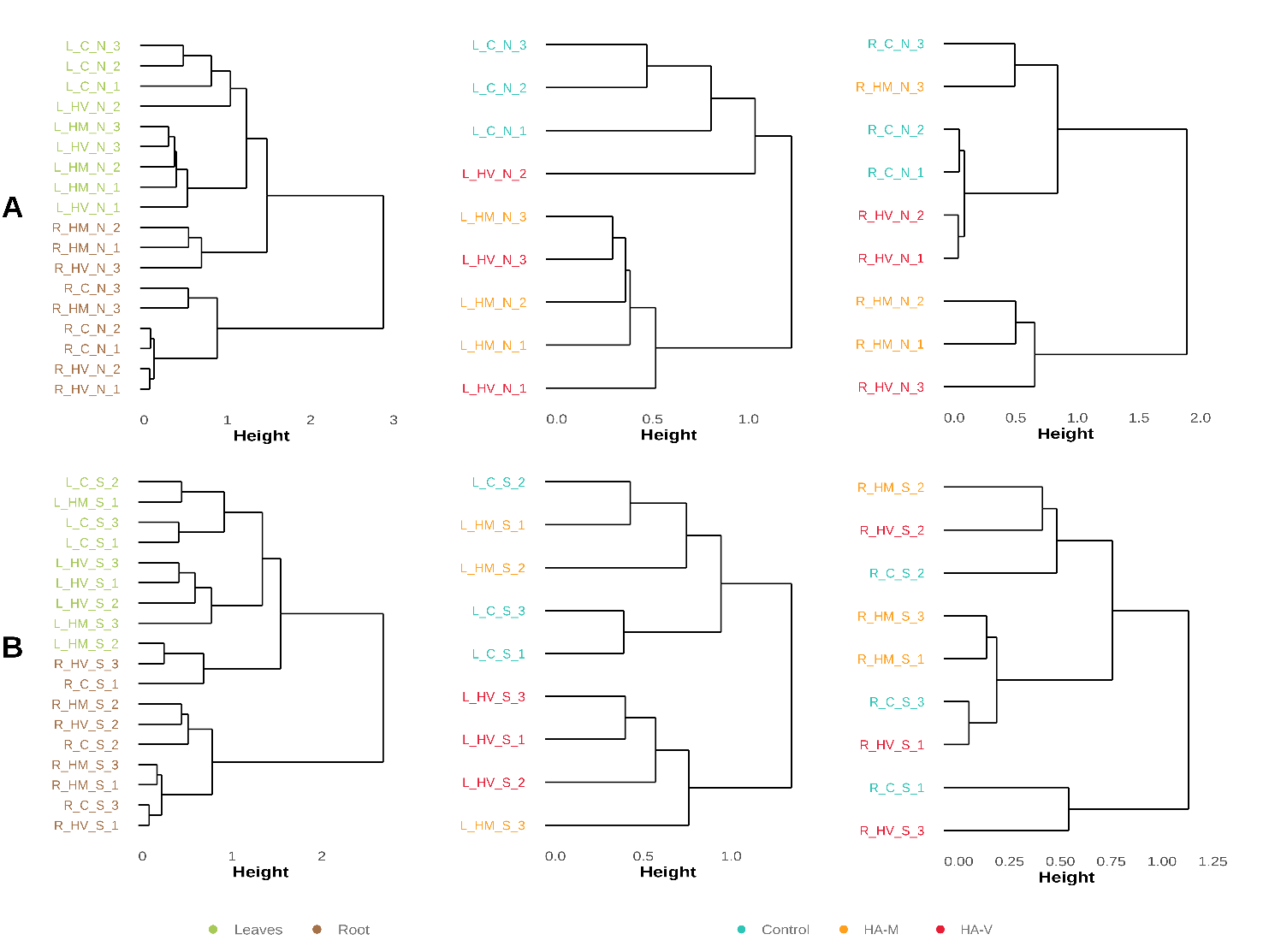 |
| --- |
| **Fig. S4.** Dendrograms of the hierarchical grouping of bacterial samples, in the absence (A) and presence (B) of stress, based on Bray‒Curtis distances. The first level (on the left) shows the dendrograms of all samples, tested in terms of separation by plant material. Then (center and right), the grouping of the leaves and roots samples, respectively, tested according to the treatments received, is observed. The treatments refer to the application of vermicompost HA (HA-V), millicompost (HA-M) and that did not receive application (Control). |

| 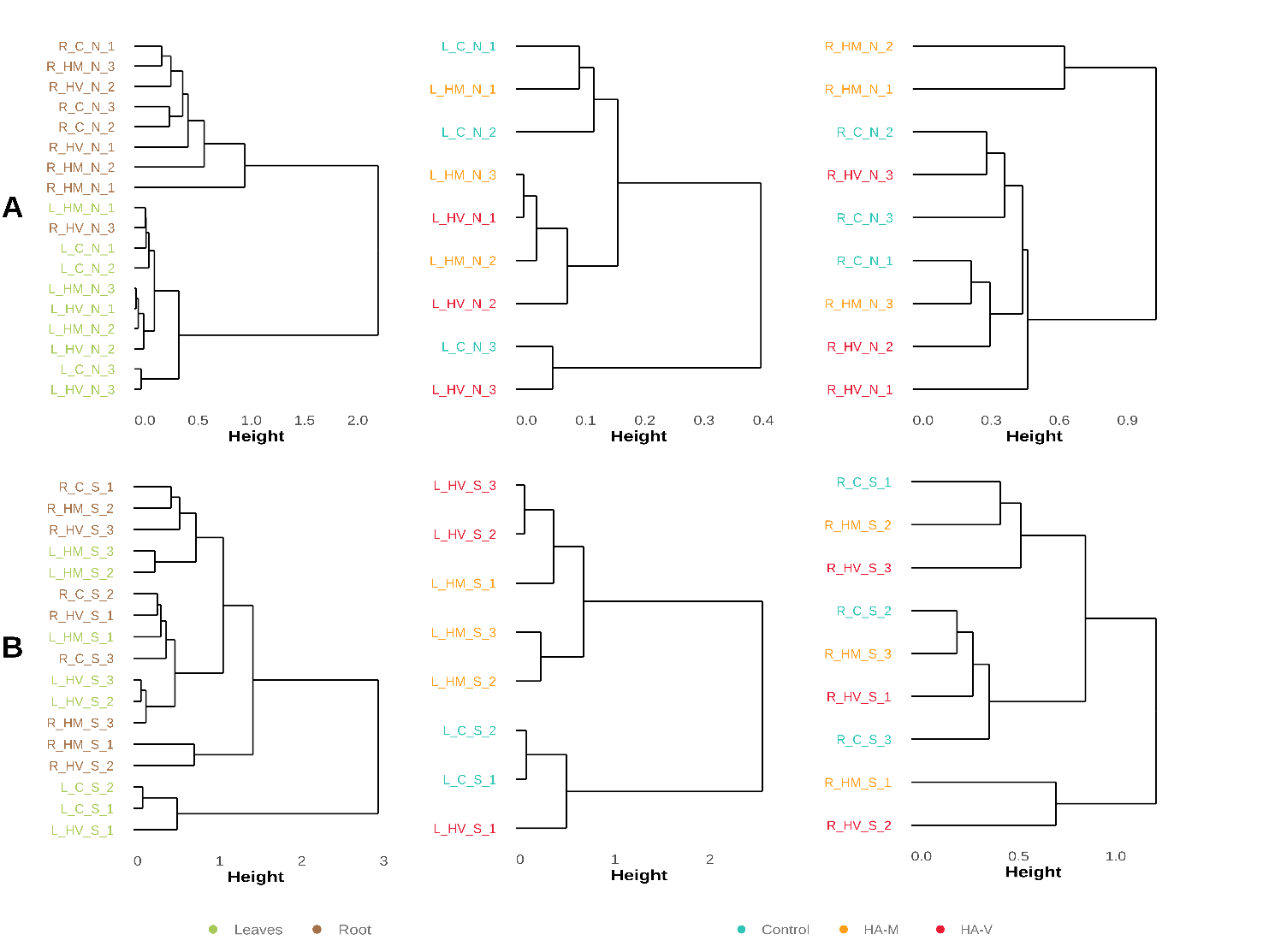 |
| --- |
| **Fig. S5.** Dendrograms of the hierarchical grouping of fungal samples, in the absence (A) and presence (B) of stress, based on Bray‒Curtis distances. The first level (on the left) shows the dendrograms of all samples, tested in terms of separation by plant material. Then (center and right), the grouping of the leaves and roots samples, respectively, tested according to the treatments received, is observed. The treatments refer to the application of vermicompost HA (HA-V), millicompost (HA-M) and that did not receive application (Control). |

| **Table S7.** Differently abundant bacterial taxa (p value < 0.05). Prefixes indicate the taxonomic level, as follows: Kingdom (k), Phylum (p), Class (c), Order (o), Family (f), Genus (g) and Species (s). The treatments (Treat.) refer to the application of vermicompost HA (HA-V), millicompost (HA-M) or that did not receive application (Control). Log2FC values indicate the number of times a taxon is more abundant in a given condition. Positive Log2FC values indicate greater abundance for the treated materials (HA-V or AHA-M) and negative values indicate greater abundance for the Control. | | | |
| --- | --- | --- | --- |
| **Taxon** | **plant material** | **Treat.** | **Log2FC** |
| *p: Bacteroidota* | Leaves | Control | -5,01 |
| *p: Firmicutes* | Leaves | Control | -3,73 |
| *c: Bacilli* | Leaves | Control | -3,26 |
| *c: Bacteroidia* | Leaves | Control | -4,17 |
| *o: Bacteroidales* | Leaves | Control | -26,63 |
| *o: Flavobacteriales* | Leaves | Control | -8,36 |
| *o: Lachnospirales* | Leaves | Control | -23,62 |
| *o: Oscillospirales* | Leaves | Control | -26,08 |
| *f: [Eubacterium] coprostanoligenes group* | Leaves | Control | -23,39 |
| *f: Bacillaceae* | Leaves | Control | -7,32 |
| *f: Bacteroidaceae* | Leaves | Control | -28,28 |
| *f: Beijerinckiaceae* | Leaves | Control | -4,60 |
| *f: Erysipelotrichaceae* | Leaves | Control | -22,44 |
| *f: Flavobacteriaceae* | Leaves | Control | -7,65 |
| *f: Labraceae* | Leaves | Control | -24,52 |
| *f: Lachnospiraceae* | Leaves | Control | -24,61 |
| *f: Oscillospiraceae* | Leaves | Control | -25,30 |
| *f: Rikenellaceae* | Leaves | Control | -24,19 |
| *f: Ruminococcaceae* | Leaves | Control | -26,27 |
| *f: Weeksellaceae* | Leaves | Control | -8,69 |
| *g: [Ruminococcus] torques group* | Leaves | Control | -24,52 |
| *g: Acinetobacter* | Leaves | Control | -4,67 |
| *g: Afipia* | Leaves | Control | -25,55 |
| *g: Alistipes* | Leaves | Control | -24,87 |
| *g: Bacillus* | Leaves | Control | -8,45 |
| *g: Bacteroides* | Leaves | Control | -29,31 |
| *g: Bosea* | Leaves | Control | -9,22 |
| *g: Brevundimonas* | Leaves | Control | -6,47 |
| *g: Chryseobacterium* | Leaves | Control | -9,59 |
| *g: Citricoccus* | Leaves | Control | -22,91 |
| *g: Faecalibacterium* | Leaves | Control | -27,54 |
| *g: Flavobacterium* | Leaves | Control | -8,49 |
| *g: Incertae Sedis* | Leaves | Control | -22,68 |
| *g: Labrys* | Leaves | Control | -25,37 |
| *g: Lachnospiraceae UCG-010* | Leaves | Control | -22,95 |
| *g: Ligilactobacillus* | Leaves | Control | -26,10 |
| *g: Lysinibacillus* | Leaves | Control | -22,66 |
| *g: Sellimonas* | Leaves | Control | -24,36 |
| *g: UCG-007* | Leaves | Control | -26,33 |
| *s: Acinetobacter_lwoffii* | Leaves | Control | -4,71 |
| *s: Brevundimonas_kwangchunensis* | Leaves | Control | -7,92 |
| *s: Flavobacterium_ummariense* | Leaves | Control | -7,85 |
| *s: Labrys_wisconsinensis* | Leaves | Control | -25,36 |
| *o: Pseudonocardiales* | Leaves | HA-M | 22,30 |
| *f: Pseudonocardiaceae* | Leaves | HA-M | 22,02 |
| *g: Amycolatopsis* | Leaves | HA-M | 21,67 |
| *g: Variovorax* | Leaves | HA-M | 8,20 |
| *o: Rickettsiales* | Leaves | HA-V | 9,49 |
| *f: Halomonadaceae* | Leaves | HA-V | 10,27 |
| *f: Morganellaceae* | Leaves | HA-V | 24,38 |
| *f: Rickettsiaceae* | Leaves | HA-V | 9,45 |
| *g: Candidatus Hamiltonella* | Leaves | HA-V | 24,41 |
| *g: Candidatus Portiera* | Leaves | HA-V | 10,16 |
| *g: Rickettsia* | Leaves | HA-V | 9,34 |
| *g: Variovorax* | Leaves | HA-V | 8,39 |
| *p: Firmicutes* | Roots | Control | -4,00 |
| *c: Bacilli* | Roots | Control | -4,14 |
| *o: Bacillales* | Roots | Control | -5,20 |
| *f: Bacillaceae* | Roots | Control | -5,47 |
| *g: Afipia* | Roots | Control | -23,37 |
| *g: Bacillus* | Roots | Control | -5,63 |
| *p: Chloroflexi* | Roots | HA-M | 5,30 |
| *c: Ktedonobacteria* | Roots | HA-M | 9,60 |
| *o: Flavobacteriales* | Roots | HA-M | 4,97 |
| *o: Ktedonobacterales* | Roots | HA-M | 8,34 |
| *f: Cellvibrionaceae* | Roots | HA-M | 20,60 |
| *f: Ktedonobacteraceae* | Roots | HA-M | 8,28 |
| *g: Cellvibrio* | Roots | HA-M | 20,51 |
| *g: Citricoccus* | Roots | HA-M | 20,72 |
|  |  |  |  |
| *g: Curvibacter* | Roots | HA-M | 20,87 |
| *g: Delftia* | Roots | HA-M | 22,07 |
| *g: Hydrogenophaga* | Roots | HA-M | 20,62 |
| *g: Knoellia* | Roots | HA-M | 19,77 |
| *g: Methylotenera* | Roots | HA-M | 21,29 |
| *g: MM2* | Roots | HA-M | 20,53 |
| *g: Pseudoduganella* | Roots | HA-M | 10,74 |
| *s: Sphingomonas_naasensis* | Roots | HA-M | 10,45 |
| *f: Sutterellaceae* | Roots | HA-V | 8,84 |
| *f: Thermomonosporaceae* | Roots | HA-V | 9,06 |
| *g: Actinomadura* | Roots | HA-V | 22,36 |
| *g: Burkholderia-Caballeronia-Paraburkholderia* | Roots | HA-V | 20,16 |
| *g: Methylobacillus* | Roots | HA-V | 8,28 |
| *g: Spirillospora* | Leaves (Stress) | Control | -8,51 |
| *g: Actinoplanes* | Leaves (Stress) | HA-M | 21,54 |
| *g: Aureimonas* | Leaves (Stress) | HA-M | 24,83 |
| *g: Candidatus Portiera* | Leaves (Stress) | HA-M | 21,35 |
| *g: Variovorax* | Leaves (Stress) | HA-M | 10,35 |
| *s: Actinoplanes_luojiashanensis* | Leaves (Stress) | HA-M | 21,73 |
| *s: Cupriavidus_pauculus* | Leaves (Stress) | HA-M | 22,27 |
| *s: Massilia_armeniaca* | Leaves (Stress) | HA-M | 21,67 |
| *s: Sphingomonas_naasensis* | Leaves (Stress) | HA-M | 21,99 |
| *g: Acinetobacter* | Leaves (Stress) | HA-V | 6,97 |
| *g: Adhaeribacter* | Leaves (Stress) | HA-V | 8,54 |
| *g: Bacillus* | Leaves (Stress) | HA-V | 5,57 |
| *g: Brevibacillus* | Leaves (Stress) | HA-V | 9,33 |
| *g: Burkholderia-Caballeronia-Paraburkholderia* | Leaves (Stress) | HA-V | 8,58 |
| *g: Candidatus Hamiltonella* | Leaves (Stress) | HA-V | 9,33 |
| *g: Candidatus Portiera* | Leaves (Stress) | HA-V | 26,08 |
| *g: Delftia* | Leaves (Stress) | HA-V | 7,14 |
| *g: Desulfonauticus* | Leaves (Stress) | HA-V | 20,89 |
| *g: Knoellia* | Leaves (Stress) | HA-V | 8,81 |
| *g: Kosakonia* | Leaves (Stress) | HA-V | 25,37 |
| *g: Neorhizobium* | Leaves (Stress) | HA-V | 8,17 |
| *g: Oxalicibacterium* | Leaves (Stress) | HA-V | 9,33 |
| *g: Paracoccus* | Leaves (Stress) | HA-V | 10,93 |
| *g: Solibacillus* | Leaves (Stress) | HA-V | 8,10 |
| *g: Stenotrophomonas* | Leaves (Stress) | HA-V | 6,27 |
| *g: Variovorax* | Leaves (Stress) | HA-V | 9,67 |
| *s: Acinetobacter_lwoffii* | Leaves (Stress) | HA-V | 8,54 |
| *s: Devosia_elaeis* | Leaves (Stress) | HA-V | 8,41 |
| *s: Sphingobium_yanoikuyae* | Leaves (Stress) | HA-V | 8,10 |
| *g: Citricoccus* | Roots (Stress) | Control | -9,73 |
| *g: Kibdelosporangium* | Roots (Stress) | Control | -24,47 |
| *s: Brevundimonas_kwangchunensis* | Roots (Stress) | Control | -7,89 |
| *s: Sphingobium_herbicidovorans* | Roots (Stress) | Control | -8,40 |
| *g: FFCH7168* | Roots (Stress) | HA-M | 21,56 |
| *g: Kosakonia* | Roots (Stress) | HA-M | 21,15 |
| *g: MN 122.2a* | Roots (Stress) | HA-M | 21,27 |
| *g: Pseudoxanthomonas* | Roots (Stress) | HA-M | 23,08 |
| *s: Chitinophaga_sedimenti* | Roots (Stress) | HA-M | 20,90 |
| *s: Novosphingobium_ginsenosidimutans* | Roots (Stress) | HA-M | 21,80 |
| *g: Actinoplanes* | Roots (Stress) | HA-V | 23,48 |
| *g: Azohydromonas* | Roots (Stress) | HA-V | 21,52 |
| *g: Hephaestia* | Roots (Stress) | HA-V | 24,19 |
| *g: Luteibacter* | Roots (Stress) | HA-V | 21,63 |
| *g: Subgroup 10* | Roots (Stress) | HA-V | 22,74 |
| *s: Actinoplanes_luojiashanensis* | Roots (Stress) | HA-V | 22,93 |
| *s: Luteibacter_jiangsuensis* | Roots (Stress) | HA-V | 21,02 |

| **Table S8.** Differently abundant fungal taxa (p value < 0.05). Prefixes indicate the taxonomic level, as follows: Phylum (p), Class (c), Order (o), Family (f), Genus (g) and Species (s). The treatments (Treat.) refer to the application of vermicompost HA (HA-V), millicompost (HA-M) or that did not receive application (Control). Log2FC values indicate the number of times a taxon is more abundant in a given condition. Positive Log2FC values indicate greater abundance for the treated materials (HA-V or HA-M) and negative values indicate greater abundance for the Control. | | | |
| --- | --- | --- | --- |
| **Taxon** | **plant material** | **Treat.** | **Log2FC** |
| *c: Dothideomycetes* | Leaves | Control | -3,27 |
| *o: Pleosporales* | Leaves | Control | -4,28 |
| *f: Erythrobasidiaceae* | Roots | Control | -9,52 |
| *o: Trichosphaeriales* | Roots (Stress) | Control | -5,94 |
| *f: Trichosphaeriaceae* | Roots (Stress) | Control | -6,23 |
| *g: Nigrospora* | Roots (Stress) | Control | -6,01 |
| *s: Nigrospora_oryzae* | Roots (Stress) | Control | -7,12 |
| *f: Clavicipitaceae* | Roots (Stress) | HA-M | 11,05 |
| *g: Balansia* | Roots (Stress) | HA-M | 24,83 |
| *g: Cercophora* | Roots (Stress) | HA-M | 21,99 |
| *s: Balansia_obtecta* | Roots (Stress) | HA-M | 24,35 |
| *s: Cercophora_mirabilis* | Roots (Stress) | HA-M | 22,58 |
| *s: Curvularia_intermedia* | Roots (Stress) | HA-M | 23,71 |
| *f: Clavicipitaceae* | Roots (Stress) | HA-V | 25,89 |
| *f: Erysiphaceae* | Roots (Stress) | HA-V | 26,49 |
| *f: Magnaporthaceae* | Roots (Stress) | HA-V | 21,54 |
| *f: Periconiaceae* | Roots (Stress) | HA-V | 7,77 |
| *g: Balansia* | Roots (Stress) | HA-V | 24,99 |
| *g: Bipolaris* | Roots (Stress) | HA-V | 24,40 |
| *g: Cercophora* | Roots (Stress) | HA-V | 24,97 |
| *g: Cladorrhinum* | Roots (Stress) | HA-V | 22,17 |
| *g: Erysiphe* | Roots (Stress) | HA-V | 26,37 |
| *g: Periconia* | Roots (Stress) | HA-V | 7,68 |
| *g: Pseudophialophora* | Roots (Stress) | HA-V | 21,47 |
| *g: Taifanglania* | Roots (Stress) | HA-V | 22,83 |
| *g: Thanatephorus* | Roots (Stress) | HA-V | 23,76 |
| *s: Balansia_obtecta* | Roots (Stress) | HA-V | 25,32 |
| *s: Cercophora_mirabilis* | Roots (Stress) | HA-V | 21,54 |
| *s: Curvularia_eragrostidis* | Roots (Stress) | HA-V | 22,38 |
| *s: Curvularia_hawaiiensis* | Roots (Stress) | HA-V | 9,67 |
| *s: Curvularia_intermedia* | Roots (Stress) | HA-V | 21,68 |
| *s: Erysiphe_diffusa* | Roots (Stress) | HA-V | 26,33 |
| *s: Periconia_epilithographicola* | Roots (Stress) | HA-V | 24,24 |
| *s: Thanatephorus_cucumeris* | Roots (Stress) | HA-V | 21,31 |

| **Table S9.** Three-way ANOVA tables for alpha diversity measurements of bacterial communities (complete model). |
| --- |
| \|  \| **Source of variation** \| **DF** \| **SS** \| **MS** \| **F value** \| **P value** \| **Sig.** \| \| --- \| --- \| --- \| --- \| --- \| --- \| --- \| --- \| \| **Chao1** \| Treatment \| 2 \| 2752 \| 1376 \| 0.72 \| 0.496 \|  \| \| Plant tissue \| 1 \| 18496 \| 18496 \| 9.72 \| 0.005 \| ** \| \| Stress \| 1 \| 8403 \| 8403 \| 4.41 \| 0.046 \| * \| \| Treatment + Plant tissue \| 2 \| 5878 \| 2939 \| 1.54 \| 0.234 \|  \| \| Treatment + Stress \| 2 \| 11181 \| 5591 \| 2.94 \| 0.072 \| • \| \| Plant_tissue + Stress \| 1 \| 11520 \| 11520 \| 6.05 \| 0.021 \| * \| \| Treatment + Plant tissue + Stress \| 2 \| 3392 \| 1696 \| 0.89 \| 0.423 \|  \| \| Residuals \| 24 \| 45685 \| 1904 \|  \|  \|  \| \| **Shannon** \| Treatment \| 2 \| 1.593 \| 0.797 \| 0.87 \| 0.431 \|  \| \| Plant tissue \| 1 \| 8.341 \| 8.341 \| 9.12 \| 0.006 \| ** \| \| Stress \| 1 \| 0.223 \| 0.223 \| 0.24 \| 0.626 \|  \| \| Treatment + Plant tissue \| 2 \| 1.251 \| 0.625 \| 0.68 \| 0.514 \|  \| \| Treatment + Stress \| 2 \| 4.75 \| 2.375 \| 2.60 \| 0.095 \| • \| \| Plant_tissue + Stress \| 1 \| 0.633 \| 0.633 \| 0.69 \| 0.414 \|  \| \| Treatment + Plant tissue + Stress \| 2 \| 1.319 \| 0.659 \| 0.72 \| 0.496 \|  \| \| Residuals \| 24 \| 21.939 \| 0.914 \|  \|  \|  \| \| **Gini-Simpson** \| Treatment \| 2 \| 0.0681 \| 0.0341 \| 0.65 \| 0.533 \|  \| \| Plant tissue \| 1 \| 0.722 \| 0.722 \| 13.68 \| 0.001 \| ** \| \| Stress \| 1 \| 0.0003 \| 0.0003 \| 0.01 \| 0.938 \|  \| \| Treatment + Plant tissue \| 2 \| 0.0383 \| 0.0192 \| 0.36 \| 0.699 \|  \| \| Treatment + Stress \| 2 \| 0.1898 \| 0.0949 \| 1.80 \| 0.187 \|  \| \| Plant_tissue + Stress \| 1 \| 0.0068 \| 0.0068 \| 0.13 \| 0.723 \|  \| \| Treatment + Plant tissue + Stress \| 2 \| 0.0649 \| 0.0324 \| 0.62 \| 0.549 \|  \| \| Residuals \| 24 \| 1.2667 \| 0.0528 \|  \|  \|  \| |
| DF = Degree of freedom; SS = Sum of squares; MS = Mean square. Significance codes: ** (p ≤ 0.01); * (p ≤ 0.05); • (p ≤ 0.1) |

| **Table S10.** Three-way ANOVA tables for alpha diversity measurements of fungal communities (complete model). |
| --- |
| \|  \| **Source of variation** \| **DF** \| **SS** \| **MS** \| **F value** \| **P value** \| **Sig.** \| \| --- \| --- \| --- \| --- \| --- \| --- \| --- \| --- \| \| **Chao1** \| Treatment \| 2 \| 255 \| 127 \| 0.24 \| 0.791 \|  \| \| Plant tissue \| 1 \| 12235 \| 12235 \| 22.77 \| >0.001 \| *** \| \| Stress \| 1 \| 0 \| 0 \| 0.00 \| 0.977 \|  \| \| Treatment + Plant tissue \| 2 \| 859 \| 430 \| 0.80 \| 0.462 \|  \| \| Treatment + Stress \| 2 \| 458 \| 229 \| 0.43 \| 0.658 \|  \| \| Plant_tissue + Stress \| 1 \| 3505 \| 3505 \| 6.52 \| 0.018 \| * \| \| Treatment + Plant tissue + Stress \| 2 \| 290 \| 145 \| 0.27 \| 0.766 \|  \| \| Residuals \| 23 \| 12357 \| 537 \|  \|  \|  \| \| **Shannon** \| Treatment \| 2 \| 0.313 \| 0.157 \| 0.65 \| 0.5305 \|  \| \| Plant tissue \| 1 \| 8.120 \| 8.120 \| 33.81 \| >0.001 \| *** \| \| Stress \| 1 \| 0.094 \| 0.094 \| 0.39 \| 0.5384 \|  \| \| Treatment + Plant tissue \| 2 \| 0.006 \| 0.003 \| 0.01 \| 0.9882 \|  \| \| Treatment + Stress \| 2 \| 0.436 \| 0.218 \| 0.91 \| 0.4178 \|  \| \| Plant_tissue + Stress \| 1 \| 1.896 \| 1.896 \| 7.90 \| 0.0099 \| ** \| \| Treatment + Plant tissue + Stress \| 2 \| 1.426 \| 0.713 \| 2.97 \| 0.0713 \| • \| \| Residuals \| 23 \| 5.524 \| 0.240 \|  \|  \|  \| \| **Gini-Simpson** \| Treatment \| 2 \| 0.0480 \| 0.0240 \| 0.84 \| 0.443 \|  \| \| Plant tissue \| 1 \| 1.0154 \| 1.0154 \| 35.68 \| >0.001 \| *** \| \| Stress \| 1 \| 0.0460 \| 0.0460 \| 1.62 \| 0.217 \|  \| \| Treatment + Plant tissue \| 2 \| 0.0047 \| 0.0024 \| 0.08 \| 0.921 \|  \| \| Treatment + Stress \| 2 \| 0.0738 \| 0.0369 \| 1.30 \| 0.293 \|  \| \| Plant_tissue + Stress \| 1 \| 0.1644 \| 0.1644 \| 5.78 \| 0.025 \| * \| \| Treatment + Plant tissue + Stress \| 2 \| 0.2117 \| 0.1058 \| 3.72 \| 0.040 \| * \| \| Residuals \| 23 \| 0.6546 \| 0.0285 \|  \|  \|  \| |
| DF = Degree of freedom; SS = Sum of squares; MS = Mean square. Significance codes: *** (p < 0.001); ** (p ≤ 0.01); * (p ≤ 0.05); • (p ≤ 0.1) |
